# FveDAD2 negatively regulates branch crowns by affecting abscisic acid metabolism through FveHB7 in woodland strawberry

**DOI:** 10.1093/hr/uhaf250

**Published:** 2025-09-17

**Authors:** Hongying Sun, Junxiang Zhang, Weijia Li, Yan Wang, Zhihong Zhang

**Affiliations:** Liaoning Key Laboratory of Strawberry Breeding and Cultivation, College of Horticulture, Shenyang Agricultural University, Shenyang 110866, China; Liaoning Key Laboratory of Strawberry Breeding and Cultivation, College of Horticulture, Shenyang Agricultural University, Shenyang 110866, China; Liaoning Key Laboratory of Strawberry Breeding and Cultivation, College of Horticulture, Shenyang Agricultural University, Shenyang 110866, China; Engineering Research Center of Coal-Based Ecological Carbon Sequestration Technology of the Ministry of Education, Shanxi Datong University, Datong 037009, China; Liaoning Key Laboratory of Strawberry Breeding and Cultivation, College of Horticulture, Shenyang Agricultural University, Shenyang 110866, China; Liaoning Key Laboratory of Strawberry Breeding and Cultivation, College of Horticulture, Shenyang Agricultural University, Shenyang 110866, China

## Abstract

The branch crown is an important trait of the strawberry that influences plant architecture and yield. Strigolactones (SLs) are significant hormones involved in the plant growth response and are crucial for regulating branching. Previous studies have shown that SL signaling regulates branching by affecting abscisic acid (ABA) biosynthesis. In this study, we observed that the SL signaling pathway can affect branching by regulating ABA catabolism in strawberry. FveDAD2 in woodland strawberry was identified as the receptor for SL. Three FveDAD2-RNAi transgenic lines exhibited the phenotype of multibranched crowns and smaller fruits. Like the alpha/beta hydrolase DWARF14 (D14), the interaction of FveDAD2 with FveSMXL7 depended on SL. The FveSMXL7-RNAi transgenic plants exhibited a less branched phenotype compared to the control plant. In addition, FveSMXL7 binds to the promoter of *FveHB7* and represses its transcription. FveHB7, a homeobox transcription factor, negatively regulates the transcription of the *ABA 8′-hydroxylase* gene (*FveABA8'OH1*). The expression of *FveHB7* was up-regulated, while the expression of the *FveABA8'OH1* was down-regulated in FveSMXL7-RNAi. ABA levels were reduced in the shoot tips of the FveDAD2-RNAi lines and increased in the FveSMXL7-RNAi lines. Treating wild-type plants with 20 μM ABA significantly suppressed the number of branch crowns, while 40 μM ABA rescued the phenotype of FveDAD2-RNAi. In conclusion, our research indicates that SL signaling may regulate branching by affecting ABA catabolism. These findings provide a theoretical basis for elucidating the mechanism of the development of branch crowns in the strawberry.

## Introduction

In strawberries (*Fragaria* spp.), the plant architecture is mainly determined by the development of the meristematic tissue. The shoot apical meristem can grow nutritionally or differentiate into a terminal inflorescence meristem. Axillary buds located in the leaf axils can remain dormant or sprout to form two shoot states: long shoots called runners, which can be used for asexual reproduction, and short shoots called branch crowns, which can form independent terminal inflorescences under suitable conditions [1]. Similar to rice tillering, which significantly affects yield, the development of the branch crown may directly impact the architecture, yield, and perennial life history of the plant [2]. Therefore, it is important to elucidate the regulatory mechanisms of the branch crown in strawberry.

Plant architecture is regulated by various endogenous and environmental signals, including the phytohormone strigolactones (SLs). SLs are a group of compounds derived from carotenoids that are critical for inhibiting branching [3]. Much has been learned about the biosynthesis and signaling of SLs by identifying and examining branching mutants in some species. The SL biosynthetic pathway seems to be conserved, but the regulation of shoot branching by the SL signaling pathway is still undetermined in higher plants.

The perception of SLs involves DWARF14 (D14), RAMOSUS3 (RMS3), and DECREASED APICAL DOMINANCE2 (DAD2). Once the receptor hydrolyzes SL, the SL is divided into the ABC-ring and D-ring [4, 5]. The receptor then covalently captures the D-ring and forms an Skp1-cullin-F-box (SCF) complex with the MAX2/D3/RMS4 F-box proteins, which leads to ubiquitination-dependent degradation of suppressor of MAX2 1-Like 6, 7, and 8 (SMXL 6,7,8) [6–8]. D53 interacts with TOPLESS (TPL) and TOPLESS-RELATED (TPR) as well as specific SQUAMOSA PROMOTER-BINDING PROTEIN-LIKE (SPL) proteins to repress the transcription of SPLs [9–11]. Additionally, SMXL6/7/8 can directly bind DNA and repress the expression of *SMXL7* in *Arabidopsis*, acting as autoregulated transcription factors (TFs) to ensure the homeostasis of SMXL6/7/8 and negatively regulate SL signaling [12].

Multiple factors, including hormones, nutrients, and light, regulate the branching of plants through the SL pathway. SMXL6/7/8 is crucial for the influence of SLs on the accumulation of PIN-FORMED (PIN) proteins and the transport of auxin, which controls bud establishment [13–15]. SLs can potentially regulate cytokinin biosynthesis and metabolism via D53/SMXL6,7,8 [16, 17]. Additionally, cytokinins enhance the expression of SMXL7/D53 in shoots [8]. SLs also affect meristem formation via D53 at different nitrogen concentrations by changing the binding of the gibberellin negative regulator SLENDER RICE1 (SLR1) to the TF GROWTH-REGULATING factor 4 (GRF4) [18]. The downstream factor regulating shoot growth of the SL repressor D53 is *TEOSINTE BRANCHED1* (*TB1*)/*FINE CULM1* (*FCL1*)/*BRANCHED1* (*BRC1*), which are highly expressed in axillary buds and inhibit bud growth [9, 19, 20]. The TB1/BRC1 pathway is linked to ABA and is important in controlling branching. Under short photoperiods, BRC1 induces *HOMEOBOX 21* (*HB21*), *HB40*, and *HB53* expression. These TFs with BRC1 further stimulate *9-CIS-EPOXICAROTENOID DIOXYGENASE 3* (*NCED3*), leading to the accumulation of ABA in shoots, which affects shoot growth [21, 22]. The TF NITROGEN-MEDIATED TILLER GROWTH RESPONSE 5 (NGR5) is involved in nitrogen-promoted tillering and is regulated through *D14* [23]. Further studies revealed that low nitrogen promotes phosphorylation and stabilization of D14, which inhibits rice tillering [24]. Sugar is an initial regulator of shoot growth in SL signaling by regulating BRC1 and cytokinins [25–27]. In rice, the light response regulator CIRCULAR CLOCK ASSOCIATED1 (CCA1) has been demonstrated to integrate sugar responses and the SL signaling pathway to repress tiller-bud outgrowth [28]. In *Arabidopsis*, FHY3 and FAR1, two homologous TFs essential for light signaling, and SMXL6/7/8, directly interact with SPL9 and SPL15, thereby suppressing their transcriptional activation of *BRC1* and promoting branching. This establishes an integrated model of light and SL coordinately regulating *BRC1* expression and branching [29].

The SL signaling pathway determines the plasticity of plant architecture in complex environments by regulating plant branching in various ways. Studies have shown that hormones such as gibberellin, auxin, and cytokinin can regulate the architecture of the strawberry, with most studies focusing on runners [1, 30, 31]. However, there are few studies on strawberry architecture in SL. In cultivated strawberries, GR24 treatment inhibits the growth of runners, and the effect on the branch crown is not apparent [32]. In this study, woodland strawberry plants without runners were selected as the material to investigate the mechanism by which SL regulates the branch crown. The FveDAD2-RNAi and FveSMXL7-RNAi transgenic lines of woodland strawberry (*Fragaria vesca*) have more or fewer branch crowns compared with wild-type (WT) plants. Our analyses on these transgenic plants confirm that the SL signaling pathway mediates ABA catabolism to regulate branch crowns in strawberry. This finding provides a new idea to improve strawberry plant architecture and yield.

## Results

### Characteristic analysis of FveDAD2

To clarify the role of SLs in regulating branch crowns in strawberry, the woodland strawberry accession ‘Yellow Wonder’ (YW), which does not typically produce runners, was treated with GR24, a synthetic analogue of SL. Treatment with 5 μM GR24 for 30 days significantly suppressed branch crown development (Fig. S1A and B). Furthermore, we generated transgenic plants that overexpress *FveD27*, which is a key gene in SL biosynthesis. Compared to the WT ‘YW’, FveD27-OE plants exhibited a significant reduction in branch crown number (Fig. S1C and D). These results demonstrate that SLs regulate branch crown formation in strawberry. To further investigate the molecular mechanisms by which SL affects strawberry branch crowns, an analysis was conducted of the receptors for SL downstream signal perception in strawberry. The amino acid sequence of AtD14, the SL receptor α/β hydrolase, was used to search for homologs in the Genome Database for Rosaceae (GDR). We identified three homologous proteins in the diploid woodland strawberry: FvH4_2g33160, FvH4_1g17110, and FvH4_1g05320, corresponding to FveDAD2, FveKAI2, and FveD14.

**Figure 1:**
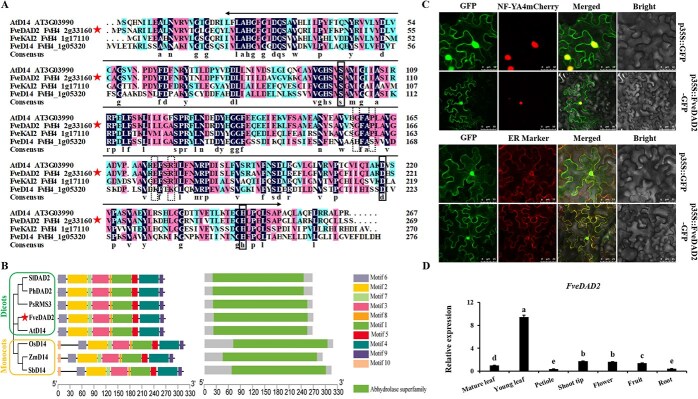
Analysis of FveDAD2 protein characteristics and *FveDAD2* gene expression patterns. (A) The amino acid sequence alignment of AtD14, FveDAD2, FveD14, and FveKAI2 was conducted using DNAMAN 5.0. FveDAD2 is highlighted by a pentagram, α/β hydrolase domain: the arrow line segment contains the sequence; catalytic triads: the solid-line box; conserved amino acid residues: the dashed-line box. (B) Phylogenetic relationship, motifs, and conserved domain analysis of FveDAD2. (C) Subcellular localization was observed for a *pro35S::FveDAD2-GFP* fusion protein in *N. benthamiana* leaves. Bar = 10/25/50 μm. (D) RT-qPCR analysis was conducted for the transcript levels of *FveDAD2* in various organs during the reproductive growth phase (DPS, Duncan’s MRT, *P* < 0.05).

The amino acid sequence identity of FveDAD2, FveKAI2, and FveD14 to AtD14 was 76.58%, 52.21%, and 39.35%, respectively (Fig. 1A). All three strawberry proteins contain a conserved α/β hydrolase domain and possess the catalytic triads (Ser97-S^AtD14^, Asp218-D^AtD14^, His247-H^AtD14^) with hydrolase characteristics [9, 33, 34]. There are four conserved amino acid residues in the reported D14. The Gly159-G^AtD14^, Pro162-P^AtD14^, and Glu175-E^AtD14^ are required for the interaction between AtD14 and D3 in *Arabidopsis* [33]. The Ser177-R^AtD14^ plays a crucial role in the binding of SsD14 to SMXL7 in sugarcane [35]. In contrast to FveDAD2 and FveKAI2, FveD14 lacks three residues: Gly159-G^AtD14^, Pro162-P^AtD14^, and Glu175-E^AtD14^. The α/β-hydrolases KARRIKIN-INSENSITIVE 2 (KAI2) and D14 are paralogous receptors for karrikins and SLs, two classes of plant growth regulators with butenolide moieties. KAI2 and D14 act in parallel signaling pathways that share a requirement for the F-box protein MAX2 but produce distinct growth responses by regulating different members of the SMAX1-LIKE/D53 family. SL primarily controls branching, while KAI2-mediated signal transduction regulates seedling establishment [36, 37]. In addition, FveDAD2 expression was significantly elevated in FveD27-OE plants, indicating that the SL receptor FveDAD2 responds to elevated SL levels (Fig. S1E and F). Therefore, in this study, FveDAD2 was selected as the receptor for SL to investigate the mechanism of the SL signaling pathway in branch crown regulation in woodland strawberry.

Phylogenetic analysis revealed a close evolutionary relationship between FveDAD2 and AtD14 (Fig. 1B). Analysis of conserved patterns and structural domains of DAD2 determined that FveDAD2 contained the nine motifs of α/β helical structures as dicots (*Solanum lycopersicum* SlDAD2, *Petunia hybrida* PhDAD2, *Pisum sativum* PsRMS3, and *Arabidopsis thaliana* AtD14), corresponding to these dicot proteins, and the N-terminal of the monocot proteins (*Oryza sativa* OsD14, *Zea mays* ZmD14, and *Sorghum bicolor* SbD14) have an additional glycine- and serine-rich domain (Fig. 1B), and contained α/β hydrolase superfamily domains in all eight model plants. The putative catalytic triad (serine, aspartate, and histidine) is necessary for SL hydrolysis, which is conserved among the three sites (Fig. S2 FveDAD2: Ser98, Asp199, His248; red arrow). Additionally, four putative crucial amino acids are used to identify components of the SCF complex (Fig. S2 FveDAD2: Gly160, Pro163, Glu176, and Ser178; green arrow). Taken as a whole, these data indicate that DAD2 is highly conserved in different plant species.

To analyze the subcellular localization of FveDAD2, *pro35S::GFP* and *pro35S::FveDAD2-GFP* were transiently expressed in the leaves of *Nicotiana benthamiana*. The *pro35S::FveDAD2-GFP* fusion protein colocalized with NF-YA4mCherry and the ER marker in the nucleus, endoplasmic reticulum, and cytoplasm (Fig. 1C; Fig. S3). To investigate the expression levels of *FveDAD2* in different organs of the woodland strawberry, we performed the quantitative RT-PCR (RT-qPCR) analysis. The results showed that *FveDAD2* was expressed in all the tested organs. The highest transcript abundance was detected in the young leaf, followed by the shoot tip, flower, fruit, mature leaf, root, and petiole (Fig. 1D).

### Silencing of *FveDAD2* increased the number of branch crowns in woodland strawberry

**Figure 2:**
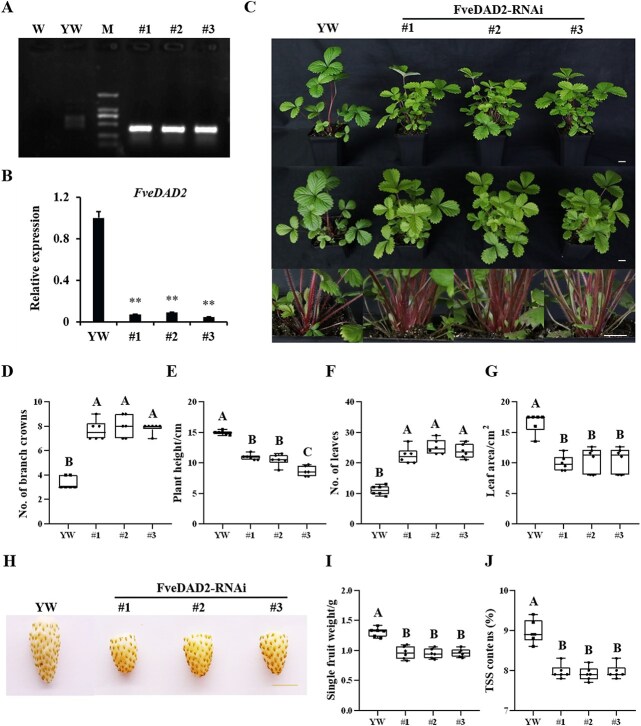
The phenotype of *FveDAD*2 silencing in ‘YW’. (A) PCR analysis of the *FveDAD2* in ‘YW’ and FveDAD2-RNAi lines. W: water; ‘YW’: nontransgenic control; M: DL2000 marker; #1, #2, #3: three independent positive FveDAD2-RNAi transgenic lines. (B) Analysis of relative expression of *FveDAD2* by RT-qPCR in ‘YW’ and FveDAD2-RNAi lines (DPS, Duncan’s MRT, ^**^P < 0.01). (C) Phenotypes of FveDAD2-RNAi lines and the ‘YW’ during the vegetative stage. Bar = 2 cm. The quantification of (D) number of branch crowns, (E) plant height, (F) number of leaves, (G) the area of the third leaf, (I) the single fruit weight, and (J) the total soluble solid contents. Values are mean ± SD (*n* = 6), A–C indicates significant differences among the plants (Prism 8.0.1, Duncan’s MRT, *P* < 0.01). (H) Phenotypes of fruit in the FveDAD2-RNAi lines and the ‘YW’ at 40 days after flowering (DAF). Bar = 1 cm.

To elucidate the role of *FveDAD2* in woodland strawberry, we utilized *Agrobacterium*-mediated transformation to introduce FveDAD2-RNAi vectors into the woodland strawberry accession ‘YW’. Three FveDAD2-RNAi lines (#1, #2, #3) were obtained. We examined these RNAi-silenced lines at the DNA level (Fig. 2A) and the RNA level. The expression of *FveDAD2* in RNAi lines was decreased to one-tenth of the ‘YW’ (Fig. 2B). The phenotypes of RNAi lines and the ‘YW’ were recorded during vegetative stages (Fig. 2C). All FveDAD2-RNAi lines had more branch crowns than ‘YW’ (Fig. 2D; Fig. S4A and B). We also identified some phenotypic differences between FveDAD2-RNAi lines and ‘YW’, and the results showed that plants of FveDAD2-RNAi lines exhibited shorter plant height, increased number of leaves, and reduced area of the third leaf (Fig. 2E–G). Thus, FveDAD2 plays a negative role in regulating branch crowns. In addition, we found that the FveDAD2-RNAi lines had significantly smaller fruits than ‘YW’ (Fig. 2H), with a 26% reduction in single fruit weight (Fig. 2I) and an 11% reduction in total soluble solids (TSS) content (Fig. 2J) in the FveDAD2-RNAi fruits compared with the fruits of ‘YW’.

### FveDAD2 and FveSMXL7, the key components of SL signaling, are structurally and functionally conserved

Following the hydrolysis of SLs by the hydrolase D14/DAD2, the activated D14 binds to MAX2/D3, thereby inducing conformational changes within the SCF complex. These changes facilitate the recognition and ubiquitination of the repressors D53/SMXL6,7,8 [8]. We hypothesize that FveDAD2 interacts with downstream repressors to transduce SL signals. *FveSMXL7* was identified by searching for homologous genes in woodland strawberry in the GDR using the rice *OsD53* mRNA sequence. This gene was found to have a high similarity of 50.2% to *AtSMXL7*.

**Figure 3:**
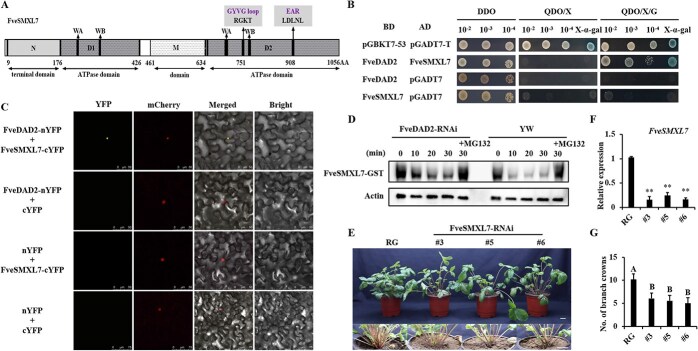
FveSMXL7, which interacts with FveDAD2, is structurally and functionally conserved. (A) The structure of the FveSMXL7 protein contains four protein domains and two essential amino acid motifs: two Walker A (WA), two Walker B (WB), a GYVG loop, and EAR motifs. (B) The Y2H assay demonstrated that FveDAD2 can interact with FveSMXL7 in the presence of GR24. The media used in this experiment included the minimal media double dropouts (DDO, SD-Leu/-Trp), minimal media quadruple dropouts (QDO/X, SD-Leu/-Trp/-His/-Ade plus X-α-gal), and (QDO/X/G, SD-Leu/-Trp/-His/-Ade plus X-α-gal and GR24). The binding domain (BD), activating domain (AD), and synthetic dropout (SD) were used. (C) BiFC assay. FveDAD2 interacts with FveSMXL7 in tobacco leaves. mCherry was used as a nuclear marker. n/cYFP, the N/C terminal of the YFP; YFP, yellow fluorescent protein. Bar = 50/75 μm. (D) *In vitro* protein degradation assay of FveSMXL7 in shoot apices of FvDAD2-RNAi and YW plants. Total protein was extracted from the shoot tips of FvDAD2-RNAi plants and WT YW plants, and proteasome inhibitor MG132 was added. The samples were incubated with the prokaryotic-expressed FveSMXL7-GST protein for 30 minutes. Actin was used as an internal reference, and GST antibody was used for Western blot analysis of the *in vitro* protein degradation of FveSMXL7. (E) Phenotypes of FveSMXL7-RNAi lines #3, #5, #6, and the ‘Ruegen’ (RG) during the early stages of reproductive growth. Bar = 2 cm. (F) Analysis of the relative expression of *FveSMXL7* by RT-qPCR in ‘RG’ and FveSMXL7-RNAi lines (DPS, Duncan’s MRT, ^**^P < 0.01). (G) Quantification of the number of branch crowns (DPS, Duncan’s MRT, *P* < 0.01).

We cloned the coding sequence (CDS) of *FveSMXL7* and performed amino acid comparisons with OsD53 and AtSMXL6/7/8. FveSMXL7 is recognized by four domains (Fig. 3A; Fig. S5): N-terminal domain, D1 ATPase domain, M domain, and D2 ATPase domain. For the D14 interaction, D1 is a necessary and sufficient condition. D2 is the key domain of receptor-induced degradation [38]. Notably, there are four protein domains, two Walker A (WA) and two Walker B (WB), in the D1 and D2 domains, which are important parts of the structural domain of the ClpB protein superfamily [6, 8]. Additionally, the D2 domain of FveSMXL7 contains a highly conserved RGKT motif, which is found in D53 and AtSMXL6/7/8 and is thought to be vital for SL-mediated D53/SMXLs protein degradation, and a highly conserved sequence, LDLNL, which is the ethylene responsive element binding factor-associated amphiphilic repression (EAR) motif, which is known to determine whether a TF is a repressor [6, 13, 39]. The results indicate that the FveSMXL7 sequence is conserved compared to D53/SMXL6,7,8 in the model plant.

**Figure 4:**
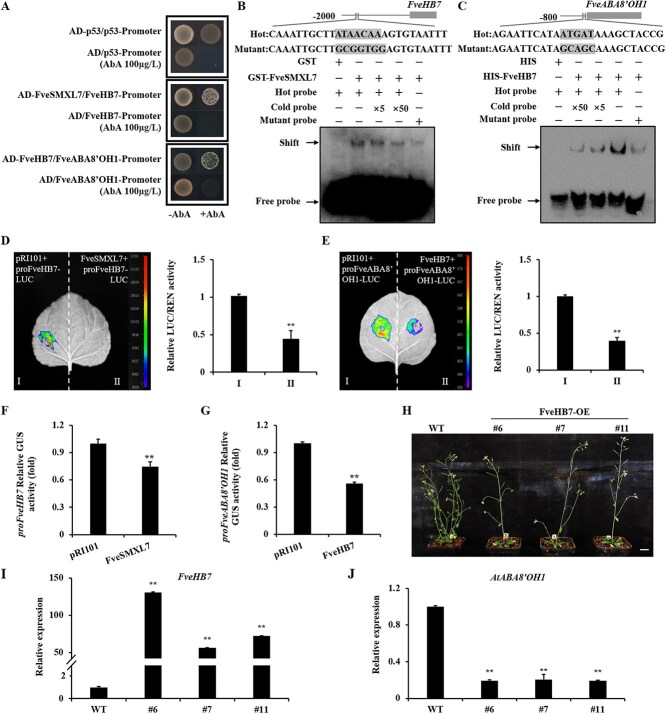
FveSMXL7 indirectly promotes *FveABA8'OH1* expression via the TF FveHB7. (A) Y1H assay indicates that FveSMXL7/FveHB7 binds to the *FveHB7*/*FveABA8‘OH1* promoter. The baseline concentration of AbA used was 100 μg/l. The positive controls utilized were AD-p53 and the p53 promoter. The negative controls utilized were the empty vector and the *FveHB7*/*FveABA8’OH1* promoter. (B and C) EMSA analysis. FveSMXL7/FveHB7 could directly bind to the sequences of the *FveHB7/FveABA8’OH1 promoters in vitro.* (D and E) Luciferase reporter assay. *pro35S::FveSMXL7/FveHB7* and *proFveHB7/FveABA8‘OH1::LUC* were cotransformed into *N. benthamiana*. (F and G) GUS activity analysis. *pro35S::FveSMXL7/FveHB7* and *proFveHB7/FveABA8’OH1::GUS* were cotransformed into *N. benthamiana* (DPS, Duncan’s MRT, ^**^P < 0.01). (H) Transgenic plants ectopically expressing *FveHB7* in *A. thaliana*, the branching phenotypes of FveHB7-OE lines #6, #7, #11, and WT. Bar = 2 cm. (I and J) Analysis of relative expression of *FveHB7* and *AtABA8’OH1* by RT-qPCR in WT and FveHB7-OE lines (DPS, Duncan’s MRT, ^**^P < 0.01).

In the presence of GR24, D53 may interact with D14 [6, 39, 40]. We investigated the interaction between FveDAD2 and FveSMXL7 by yeast two-hybrid (Y2H) and observed physical interactions between them. This interaction was dependent on GR24 (Fig. 3B; Fig. S6A). Bimolecular fluorescence complementation (BiFC) assays verified that FveDAD2 interacted with FveSMXL7 in the nucleus (Fig. 3C). *In vitro* SMXL7 protein degradation assays showed the degradation capacity of FveSMXL7 in the shoot apices of FveDAD2-RNAi plants was significantly reduced compared to ‘YW’ (Fig. 3D). This suggests that the protein levels of FveSMXL7 in the shoot apices of FveDAD2-RNAi plants are higher than those in the WT.

In addition, we obtained three FveSMXL7-RNAi lines (Fig. 3E) in the background of the woodland strawberry accession ‘Ruegen’ (RG), whose fruits are red. We found that the expression of *FveSMXL7* in the RNAi lines was reduced to a quarter of that in ‘RG’ (Fig. 3F). Compared with the ‘RG’, all the FveSMXL7-RNAi lines presented fewer branch crowns during the early stages of reproductive growth (Fig. 3G). These results indicate that FveDAD2 and FveSMXL7, as members of the SL signaling pathway, are structurally and functionally conserved, providing a solid foundation for exploring downstream components of SL-regulated branch crowns in strawberry.

### ABA may be involved in regulating branching by *FveDAD2*

To find the reason for *FveDAD2* regulating branch crowns in the strawberry, we performed transcriptome analysis of shoot tips in FveDAD2-RNAi and ‘YW’. Volcano plots showed that there were 204 different expression genes (DEGs; |Fold Change| ≥ 1.5 and FDR < 0.05). Among them, 106 were up-regulated and 98 were down-regulated in FveDAD2-RNAi plants compared to ‘YW’ (Fig. S7A). DEGs are enriched in plant hormone signaling, starch, and sucrose metabolism according to KEGG pathway analysis (Fig. S7B). Next, we referred to the multidatabase annotations of DEGs and screened 34 hormone- and sugar-related genes among all DEGs, including 22 hormone-related genes and 12 sugar-related genes (Table S1). Hormone-related genes accounted for a relatively large proportion of genes involving hormones such as ABA (8), auxin (3), BR (1), CTK (3), ETH (1), GA (4), and SL (2), among which ABA-related differential genes were the most numerous. Therefore, it is hypothesized that in woodland strawberry shoot tips, multiple phytohormones may be involved in the FveDAD2 regulation of branch crowns, especially ABA.

### FveSMXL7 indirectly promotes *FveABA8'OH1* expression through the TF FveHB7

The transcriptional target of the SL pathway for shoot growth regulation, *TB1/FCL1/BRC1*, is linked to ABA. *BRC1* promotes the expression of three TFs (HB21, HB40, and HB53), which stimulate the expression of *NCED3*, resulting in ABA accumulation and subsequently affecting meristematic conditions [21]. To further investigate the molecular mechanisms by which the SL signaling pathway may cross-talk with ABA in regulating the branch crown of woodland strawberry, we analyzed the transcriptomes of FveDAD2-RNAi and ‘YW’ for ABA-related DEGs. However, these components (*BRC1* and *NCED3*) were not found in our transcriptome data. It is noteworthy that two additional genes were identified as being associated with ABA (Table S1). One down-regulated gene encodes a TF, the homeodomain leucine zipper (HD-Zip) class I HB7, which has a crucial function in the ABA signaling pathway [41–44]. And one up-regulated key ABA catabolic enzyme, *abscisic acid 8'-hydroxylase 1* (*ABA8’OH1/CYP707A*), which modulates endogenous ABA homeostasis in many plants [45–47]. It was hypothesized that the FveDAD2 and FveSMXL7 may regulate branch crowns by affecting ABA catabolism.

D53/SMXL6,7,8 are transcriptional regulators that lack direct DNA binding ability, indicating that they require adaptors to bind DNA to facilitate SL-regulated transcription and shoot branching [48]. D53 interacts through the EAR motif with TPR2 to recruit TFs that regulate SL signaling and shoot branching. We examined the interactions between FveSMXL7 and FveHB7 by Y2H (Fig. S6B) and found that they did not interact. Moreover, it was found that SMXL6 and SMXL7 can directly bind to promoters with ATAACAA motifs and negatively regulate gene expression [12]. We analyzed the promoters of *FveHB7* and *FveABA8'OH1*, and only one possible binding motif, ATAACAA, was found at 1642 bp upstream of the *FveHB7* transcription start site. Y1H analysis showed that FveSMXL7 binds to the promoter of *FveHB7* (Fig. 4A), but not to the promoter of *FveABA8'OH1* (Fig. S8A). Electrophoretic mobility shift assay (EMSA) analyses further revealed that FveSMXL7 binds to the ATAACAA sequence within the *FveHB7* promoter (Fig. 4B), which decreases with increasing competitor levels. The transcriptome data showed that the expression level of *FveHB7* was significantly down-regulated in the shoot tips of the FveDAD2-RNAi plants (Table S1). We next examined the effect of FveSMXL7 on the transcription of *FveHB7* utilizing the luciferase (LUC) reporter system and the β-glucuronidase (GUS) transactivation assay in *N. benthamiana* leaves. When *pro35S::FveSMXL7* was cotransformed with *proFveHB7::LUC*, the LUC signal was significantly weakened or even disappeared compared with the control (Fig. 4D). When *pro35S::FveSMXL7* was cotransformed with *proFveHB7::GUS*, GUS activity demonstrated a significant reduction in comparison to the control (Fig. 4F). These data show that FveSMXL7 repressed the expression of *FveHB7*.

We hypothesize that FveHB7 affects ABA levels through *FveABA8'OH1*. The HD-Zip family TFs can bind to a specific DNA sequence, CAAT(A/T)ATTG, and promote gene expression [49–51]. However, we did not find the CAAT(A/T)ATTG site for FveHB7 in the promoter fragment of *FveABA8'OH1*. In tomatoes, the HD-zip family TF, SlHB15A, has been discovered to bind to an ATGAT DNA motif, which alters the cross-talk between auxin, jasmonic acid (JA), and ethylene, which regulates flower pedicel abscission and fruit set [52, 53]. As expected, we found three possible binding sites (ATGAT) at −595, −205, and −82 bp in the promoter of *FveABA8'OH1*. We cloned the promoter of *FveABA8'OH1* with a length of 742 bp and conducted Y1H assays. The Y1H results showed that FveHB7 can bind to the promoter of *FveABA8'OH1* (Fig. 4A). Furthermore, the segmented Y1H (Fig. S8B) and EMSA analyses proved that FveHB7 binds to sequences containing ATGAT (−82 bp) in the *FveABA8'OH1* promoter (Fig. 4C). Further, LUC and GUS assays (Fig. 4E and G) show that FveHB7 can bind to the promoter of *FveABA8'OH1* and repress the expression of *FveABA8'OH1*.

The above results indicate that FveSMXL7 binds to the *FveHB7* promoter and negatively regulates its expression, whereas FveHB7 binds to the *FveABA8'OH1* promoter and represses its transcriptional activity. This regulatory cascade indicates that FveSMXL7 indirectly enhances *FveABA8'OH1* expression by suppressing the transcript abundance of *FveHB7*. To elucidate the role of the FveSMXL7-FveHB7-FveABA8'OH1 module in woodland strawberry, the *FveHB7* gene was ectopically expressed in *A. thaliana*, and *FveHB7*-overexpressing plants exhibited a reduced number of branches as compared to the WT (Fig. 4H). Compared with those in WT, the expression level of *FveHB7* was up-regulated, and the expression level of the *AtABA8'OH1* was down-regulated in FveHB7-OE lines (Fig. 4I and J). These results suggest that the SL signaling pathway may regulate branch crowns by affecting the ABA content in the shoot tips of woodland strawberry.

### ABA levels were changed in the shoot tips of the FveDAD2-RNAi and FveSMXL7-RNAi lines

**Figure 5:**
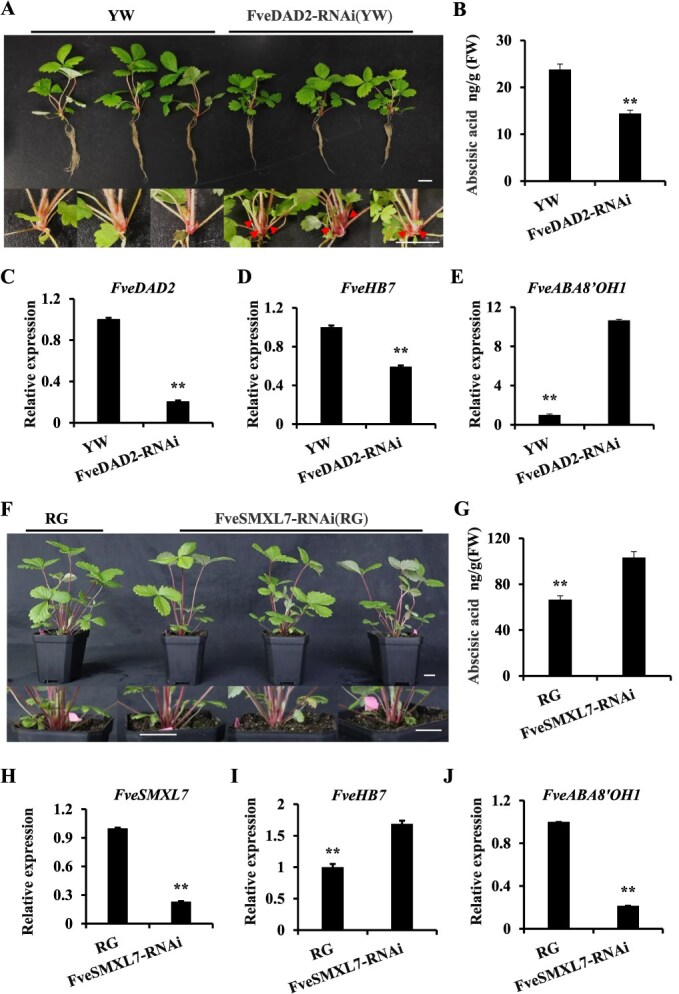
Abscisic acid was detected in the shoot tips of FveDAD2/FveSMXL7-RNAi lines and woodland strawberry. (A) Plant morphology in phytohormone assays using live seedlings grown for 60 days under comparable conditions. Bar = 1 cm. (B) Abscisic acid (ABA) levels in the shoot tips of FveDAD2-RNAi lines and ‘YW’. FveSMXL7-RNAi lines. (C–E) Analysis of relative expression of *FveDAD2*, *FveHB7*, and *FveABA8’OH1* by RT-qPCR in ‘YW’ and FveDAD2-RNAi lines (DPS, Duncan’s MRT, ^**^P < 0.01). (F) Plant morphology in phytohormone assays using live seedlings grown for 75 days under comparable conditions. Bar = 2 cm. (G) ABA levels in the shoot tips of FveSMXL7-RNAi lines and ‘RG’. (H–J) Analysis of relative expression of *FveSMXL7*, *FveHB7*, and *FveABA8’OH1* by RT-qPCR in ‘RG’ and FveSMXL7-RNAi lines (DPS, Duncan’s MRT, ^**^P < 0.01).

To verify whether ABA is associated with the regulation of strawberry branch crowns, the ABA levels in the shoot tips of both FveDAD2-RNAi lines and nontransgenic plants were analyzed in 60-day-old plants under comparable growth conditions. The FveDAD2-RNAi lines exhibited 2–3 branch crowns (red arrow), whereas the ‘YW’ lines did not present branch crowns. The transgenic lines exhibited a distinct branching and dwarfing phenotype in contrast to the ‘YW’ (Fig. 5A). We found that the level of ABA in FveDAD2-RNAi decreased by 39.6% compared to that in ‘YW’ (Fig. 5B). Compared with those in ‘YW’, the expression levels of *FveDAD2* and *FveHB7* were down-regulated, and the expression level of the *FveABA8'OH1* was up-regulated in FveDAD2-RNAi (Fig. 5C–E), which is consistent with the transcriptome data (Table S1). When the FveSMXL-RNAi transgenic lines exhibited a reduced branching phenotype compared to ‘RG’ (Fig. 5F), the level of ABA in the FveSMXL7-RNAi plants was 1.56 times higher than in the ‘RG’ plants (Fig. 5G). Compared with those in ‘RG’, the expression levels of *FveSMXL7* and *FveABA8'OH1* were down-regulated, and the expression level of *FveHB7* was up-regulated in FveSMXL7-RNAi (Fig. 5H–J). These findings provide evidence that the reduction in ABA levels leads to a multibranched phenotype in FveDAD2-RNAi lines.

To further determine the regulatory effect of ABA on strawberry branch crowns, we treated woodland strawberry ‘YW’ with ABA and fluridone (an inhibitor of ABA) for 1 month. The number of crown branches significantly decreased in the 20 μM ABA-treated ‘YW’ plants, whereas the number of crown branches increased in the 75 μM fluridone-treated ‘YW’ plants (Fig. S9A and B). Significant differences in leaf number and area were also detected among the different treatments (Fig. S9C and D). Given the reduced ABA levels in FveDAD2-RNAi, we continued to treat the shoot tips of FveDAD2-RNAi with ABA (Fig. S9E). Compared with those in the control (water treatment), the number of branch crowns was reduced to four after 20 μM ABA treatment, and there was little difference in the number of leaves and leaf area. Upon application of 40 μM ABA, the number of branch crowns was reduced to three, and the number of leaves and leaf area were comparable to those of the control (Fig. S9F–H). Therefore, it can be concluded that exogenous application of sufficient ABA could rescue the multibranched phenotype of FveDAD2-RNAi. These data further support the idea that the ABA content in shoot tips is a negative regulator of strawberry branch crowns.

## Discussion

### SL regulates plant branching by cross-talking with ABA

SLs and ABA are both derived from all-trans-β-carotene. In contrast to SLs, which inhibit plant branching through multiple pathways, studies of ABA regulation of meristems have focused on the relationship between ABA content in shoot buds and bud dormancy. In *Arabidopsis*, BRC1 has been shown to promote the expression of genes involved in ABA biosynthesis, and an increase in ABA levels in buds has been observed to result in a suppression of bud development [21]. In rice, the expression of the ABA biosynthesis gene, *OsNCED1*, has been observed to be specifically up-regulated in dormant axillary buds. The overexpressing *OsNCED1* plants accumulated extremely high levels of ABA and formed fewer tillers, thus suggesting an important role for ABA in inhibiting shoot growth [54]. In addition, studies have demonstrated that SLs can activate *BRC1* transcription, induce *HB40* expression, and promote ABA biosynthesis in shoots, thereby affecting branching [12]. This further corroborates the notion that SLs can regulate branching by affecting ABA biosynthesis. However, it is not clear whether SL regulation of branching involves ABA catabolism.

Exogenous application of ABA to rice suppresses the multibranching phenotype of SL-associated mutants *d10* and *d14* [54], which is consistent with our findings that moderate amounts of ABA can reduce the number of branches (Fig. S9F) in FveDAD2-RNAi transgenic plants. It has been shown that *rac*-GR24 treatment induces the expression of ABA biosynthesis-related genes at the base of rice stems and promotes increased endogenous ABA levels [55]. However, the absence of differential genes related to ABA synthesis in the FveDAD2-RNAi transcriptome may indicate that the induction of ABA biosynthesis genes by SL is attenuated in FveDAD2-RNAi plants. In comparison with the WT, higher expression of *FveSMXL7* (Table S1) and *FveABA8'OH1* (Fig. 5E), and reduced shoot tip ABA content were observed in multibranched FveDAD2-RNAi transgenic plants (Fig. 5A and B). Conversely, in the less branched FveSMXL7-RNAi plants, the expression of the *FveABA8'OH1* (Fig. 5J) was reduced, and the ABA content in the shoot tip was increased (Fig. 5F and G). Furthermore, the loss of *CsCYP707A4* (the *ABA 8′-hydroxylase* gene) function has been demonstrated to increase ABA levels and to inhibit axillary bud outgrowth in cucumbers [56]. It was speculated that SL signaling in strawberry might regulate branch crowns through ABA metabolism; however, Y1H results showed that FveSMXL7 did not bind to the promoter of *ABA8'OH1* (Fig. S8A). There may be a mediator between SL signaling and ABA metabolism. FveHB7, along with the intermediate component of SL-regulated ABA biosynthesis, HB40, are from the HD-Zip family [21]. Our findings demonstrate that FveSMXL7 can indirectly promote *FveABA8'OH1* expression through the TF FveHB7 (Fig. 4) and that the number of branches is significantly reduced in plants ectopically expressing *FveHB7* in comparison with the WT. Taken together, we identified a novel pathway for SL signaling to regulate branch crowns through ABA metabolism.

### SL may respond to water stress by cross-talking with ABA

Both SL and ABA have been identified as pivotal hormones that regulate developmental processes and modulate responses to various abiotic stresses [57, 58]. In particular, evidence of substantial SL and ABA cross-talk has been demonstrated in plant responses to drought stress. The application of GR24 in a practical production context has been shown to enhance crop stress tolerance by decreasing stomatal opening whilst increasing chlorophyll and ABA levels by affecting photosynthesis, transpiration, and yield [59, 60]. SLs regulate stomatal conductance and reduce transpirational water loss by promoting stomatal closure. This finding suggests that SL is an important regulator of stomatal opening, similar to ABA [61]. Negative regulators of SL signaling (SMXL1, 6, 7, and 8) affect drought tolerance; overexpression of *MsSMXL1* increases sensitivity to water-deficient environments, and *smxl6*, *7*, *8* mutants enhance drought tolerance in *Arabidopsis* [62, 63]. Loss-of-function analysis of the *Arabidopsis D14* gene showed that *d14* mutant plants were more susceptible to drought stress than WT plants, which was associated with their larger stomatal aperture and slower ABA-mediated stomatal closure under drought stress [36]. A complex interaction between SL signaling and ABA signaling pathways under drought conditions was revealed.

The AtHB7 TF is categorized as part of the homologous structural domain-leucine zip subfamily I (HD-Zip I), which has been implicated in the control of plant development and abiotic stress responses [64]. In addition, the *McHB7* overexpressing plants can increase abscisic acid levels to improve plant drought tolerance [65]. Our study demonstrated the efficacy of the FveSMXL7-FveHB7-FveABA8’OH1 module in the regulation of plant branching development. Furthermore, the *FveHB7* was found to link SL signaling with ABA metabolism, providing a novel perspective for future research on the synergistic effects of SLs and ABA in enhancing drought tolerance in plants.

### *FveDAD2* and *FveSMXL7* can be candidate target genes for the genetic improvement of strawberry plants and fruit size

Plant branching is inextricably linked to yield, and the multibranched phenotype of FveDAD2-RNAi transgenic plants manifests not only during the vegetative period but also during the reproductive period (Fig. S4A–C). Compared to the WT, transgenic corollas were smaller in diameter (Fig. S4F and G), produced smaller fruits, weighed less per fruit (Fig. 3H and I), and had significantly lower soluble solids content (Fig. 3J). Studies on the expression of SL-related genes in strawberry fruit at different developmental stages may explain this phenotype. SL pathway genes are highly expressed during early fruit development, but expression of these genes decreases or is absent as the fruit develops and matures, suggesting that SL is involved in the early development of strawberry fruit [66], which is in line with our results. The 5-deoxystrigol level was reduced in the shoot tips of FveDAD2-RNAi lines (Fig. S4D). The expression level of *FveD27* (Fig. S4E) was reduced in the FveDAD2-RNAi transgenic plants, and it was hypothesized that SL might affect the development of strawberry fruits during the green fruit expansion period. The reduction of *FveD27* in FveDAD2-RNAi lines may be attributed to a negative feedback regulatory mechanism, whereby redundant SL in the plant causes a reduction in the synthetic gene *FveD27* due to a reduction in SL-sensing hydrolysis in FveDAD2-RNAi plants to maintain hormonal homeostasis [67, 68]. In addition, the *FveABA8'OH1* homolog, *FveCYP707A4a*, has been demonstrated to regulate the fruit size and ripeness through the control of ABA levels and the coordination of auxin, GA, and ABA signaling [69]. The reduced fruit size phenotypes of the FveDAD2-RNAi plants indicate that SL may participate in the complex network of multiple hormones that regulate plant branching and fruit morphology. *FveDAD2* and *FveSMXL7* may be candidate target genes for the genetic improvement of strawberry plants and fruit size.

**Figure 6:**
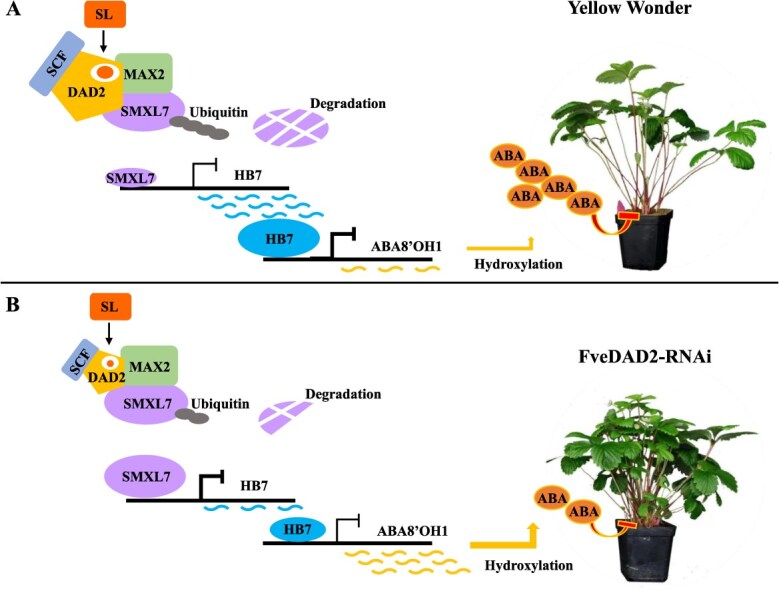
The model of SL signaling to regulate branch crowns by affecting ABA metabolism in the woodland strawberry. (A) ‘YW’, (B) FveDAD2-RNAi line.

In conclusion, we propose a model for regulating the branch crowns by FveDAD2 in woodland strawberry. In WT plants, FveDAD2 interacts with FveSMXL7 and ubiquitinates FveSMXL7 (Fig. 6A), whereas down-regulation of *FveDAD2* results in reduced ubiquitination of FveSMXL7, leading to increased repression of *FveHB7* transcription by FveSMXL7. This repression also results in reduced negative regulation of *FveABA8'OH1* by FveHB7. The increased *FveABA8'OH1* transcript level decreased ABA accumulation in the shoot tips, ultimately increasing the number of branch crowns (Fig. 6B). This model contributes to our understanding of SL-mediated ABA metabolism regulation of branch crowns in woodland strawberry, refines the theory of branch development, and lays a foundation for breeding high-yield strawberry cultivars.

## Materials and methods

### Phylogeny and sequence analysis

Protein sequences of *A. thaliana* D14 (AtD14) and *F. vesca* DAD2/D14/KAI2(FveDAD2/FveD14/FveKAI2) were retrieved from the *Arabidopsis* Information Resource (TAIR) (https://www.arabidopsis.org/) and the GDR (https://www.rosaceae.org/), respectively. These sequences were compared using DNAMAN 5.0 software. Homologous protein sequences for FveDAD2 from model species such as *S. lycopersicum* DAD2 (SlDAD2), *P. hybrida* DAD2 (PhDAD2), *P. sativum* RMS3 (PsRMS3), *Z. mays* D14 (ZmD14), *S. bicolor* D14 (SbD14), and *O. sativa* D14 (OsD14) were obtained from the National Center for Biotechnology Information (NCBI) website (https://www.ncbi.nlm.nih.gov/). The phylogenetic tree was constructed using TBtools 2.202 and MEGA 12 software. The neighbor-joining (NJ) method was used to construct the tree with bootstrap analysis with 1000 replications. Conserved motifs and domains were analyzed and plotted using TBtools 2.202 software, the MEME online website (https://meme-suite.org/meme/doc/meme.html), and the NCBI Conserved Domain Search website (https://www.ncbi.nlm.nih.gov/Structure/cdd/wrpsb.cgi).

### Plant materials and treatments

The diploid woodland strawberry (*F. vesca*) ‘YW’ was cultured under greenhouse conditions (16 h/8 h, light/dark, 23°C) at Shenyang Agricultural University, China, using substrates (the volume ratio of grass charcoal, vermiculite, earthworm feces and perlite was 4:2:2:1). Different organs were harvested simultaneously for gene expression analysis during early fruit ripening, frozen in liquid nitrogen, and stored at −80°C.

Transgenic lines of FveDAD2-RNAi in the ‘YW’ background and FveSMXL7-RNAi in the ‘Ruegen’ (RG) background were generated from young tissue-cultured woodland strawberry plantlets via *Agrobacterium*-mediated leaf disc transformation [70].

*Nicotiana benthamiana* plants grown under a long-day photoperiod (16 h/8 h, light/dark) at 23°C for 30–50 days were used for transient expression analysis.

Transgenic *Arabidopsis* lines ectopically expressing *FveHB7* were generated via floral dip transformation using *A. thaliana* (Columbia). Seeds of both transgenic lines and WT were vernalized at 4°C for 2 days, then germinated and grown at 22°C under long-day conditions (16 h light/8 h dark).

Uniform 50-day-old *F. vesca* ‘YW’ seedlings grown in potting substrate were used. Plants were at the prebranching stage with consistent growth conditions and homogeneous phenotypes. Experimental treatments included the following: abscisic acid (ABA, CAS: 21293-29-8; Coolaber, Beijing, China) at concentrations of 5, 10, 20, and 40 μM; fluridone (ABA biosynthesis inhibitor, CAS: 59756-60-4; Coolaber) at 25, 50, and 75 μM; and control group treated with deionized water. Each treatment group contained five plants. Using a micropipette, 500 μl of treatment solution was slowly applied to the central growing point of each plant. Applications were performed twice weekly for four consecutive weeks. Identical procedures were applied to 50-day-old FveDAD2-RNAi lines cultivated under equivalent conditions.

### Gene expression analyses

Gene expression was analyzed by quantitative RT-PCR using SYBR^®^ Green II (Takara, Dalian, China) on an ABI7500 Real-Time PCR System (Applied Biosystems, Foster City, CA). Three independent biological replicates were performed for each sample, with normalization against the Fve26S reference gene [70]. Relative transcript levels were calculated by the 2^–ΔΔCT^ method. The primers used are shown in Table S2.

### Subcellular localization

The CDS of FveDAD2 (excluding the stop codon) was cloned into the pRI101-GFP vector to generate the *pro35S::FveDAD2-GFP* fusion construct. The *pro35S::GFP* and *pro35S::FveDAD2-GFP* plasmids were transformed into *Agrobacterium tumefaciens* strain GV3101, respectively. Bacterial resuspensions (OD_600_ = 1) harboring these constructs were co-infiltrated with organelle markers (NF-YA4-mCherry [71] and endoplasmic reticulum marker) into leaves of 40-day-old *N. benthamiana* plants. After 48 h of dark adaptation, GFP fluorescence was examined using a confocal laser scanning microscope (Leica DMi8 A, Wetzlar, Germany). Cloning primers are provided in Table S2.

### Transcriptome sequencing analysis

Shoot tips of ‘YW’ and FveDAD2-RNAi plants were collected separately for transcriptome sequencing, when significant differences in new branch crown number became apparent between WT and transgenic plants, during the nutrient growth period (70 days old). Three biological replicates were performed. The samples were subsequently sent to the Illumina sequencing platform at Biomarker Technologies (Beijing, China). Differentially transcribed genes were screened using a threshold of |Fold Change| ≥ 1.5 and FDR < 0.05. The genes were then functionally classified and analyzed for differential expression.

### Y2H assay

Protein interactions between FveDAD2 and FveSMXL7 were verified using the GAL4-based Y2H system. The CDS of *FveDAD2* was cloned into the pGBKT7 vector, and the CDS of *FveSMXL7* was inserted into the pGADT7 vector. The recombinant plasmids were co-transformed into yeast strain Y2H using the *Saccharomyces cerevisiae* competent cell preparation and transformation kit (Protein Interaction Bio, Wuhan, China). The positive clones were screened on SD/-Trp/-Leu agar medium, SD/-Trp/-Leu/-His/-Ade/X-α-Gal agar medium, and SD/-Trp/-Leu/-His/-Ade/X-α-Gal/GR24 agar medium. GR24 (CAS: 76974-79-3; Yuanye, Shanghai, China) dissolved in dimethyl sulfoxide (DMSO) was added to the medium at a final concentration of 5 μM. Protein interactions were assessed by incubation for 72 h at 30°C. Primer sequences are shown in Table S2.

### BiFC assay

The CDS of FveDAD2 with the stop codon removed was linked to the pSPYNE vector containing the N-terminal of the sequence encoding YFP (nYFP) to create the FveDAD2-nYFP vector. The CDS of FveSMXL7 without the stop codon was linked to the pSPYCE vector with the C-terminal of the sequence encoding YFP (cYFP) to create the FveSMXL7-cYFP vector. *A. tumefaciens* strain GV3101 strains harboring these constructs were resuspended to OD_600_ = 1, mixed at a 1:1 ratio, and co-infiltrated into abaxial surfaces of the 40-day-old *N. benthamiana* leaves. After 48-h dark incubation at 22°C, reconstituted YFP signals were detected using the confocal fluorescence microscopy (Leica DMi8 A, Wetzlar, Germany). The corresponding primers are listed in Table S2.

### Y1H assay

The Y1H assay confirmed that FveSMXL7 binds to the promoter of *FveHB7*, and FveHB7 binds to the promoter of *FveABA8'OH1*. The CDS of *FveSMXL7/FveHB7* was individually cloned into the pGADT7 vector to generate the prey construct. The *FveHB7* promoter fragment (1715 bp) and the *FveABA8'OH1* promoter fragment (742 bp) were inserted into the linearized pAbAi vector, respectively, to generate bait constructs. The bait construct and the corresponding prey construct were sequentially transformed into yeast strain Y1HGold using the *S. cerevisiae* competent cell transformation kit (Protein Interaction Bio, Wuhan, China). The transformants were screened on SD/-Leu medium containing 100 ng/mL aureobasidin A (AbA). After incubation at 30°C for 72 h, interactions were confirmed by growth assay. The primers used are shown in Table S2.

### Luciferase reporter assay

To generate the effector vectors *pro35S::FveSMXL7* and *pro35S::FveHB7*, the CDS of *FveSMXL7* was ligated into the pRI101 vector, and the CDS of *FveHB7* was inserted into the pRI101 vector. The *FveHB7*/*FveABA8'OH1* promoter sequence (2000 bp), respectively, was cloned into the pGreenII0800-LUC vector to construct the reporter vector. The *A. tumefaciens* strain GV3101(pSoup) mediated the co-infiltration of the corresponding vectors into the leaves of 50-day-old *N. benthamiana* plants. After 48 h, LUC signaling was observed using an intravital fluorescence imager (Lb985, Berthold, Germany), and quantitative analysis was performed using a LUC assay kit (E1910; Promega, Madison, WI). The primers used are shown in Table S2.

### GUS analysis

The effector vectors *pro35S::FveSMXL7* and *pro35S::FveHB7* were constructed (refer to the luciferase reporter assay). *FveHB7/FveABA8'OH1* promoter sequences (2000 bp) were cloned into pRI201-GUS to generate the reporter vectors. The *A. tumefaciens* strain GV3101 mediated the co-infiltration of corresponding vectors into the leaves of the 50-day-old *N. benthamiana* plants, and GUS activity was measured as described previously [72]. The primers used are shown in Table S2.

### Recombinant protein purification and EMSAs

The full-length CDS of *FveSMXL7* were cloned and inserted into the expression vector pGEX 6p-1. The GST and GST-FveSMXL7 proteins were induced using 0.2 mM isopropyl-β-d-thiogalactopyranoside (IPTG) at 16°C for 20 h in BL21 transit cells (AngYuBio, Shanghai, China). The fusion proteins were purified with ProteinIso^®^ GST resin (TransGen, Beijing, China). The CDS of *FveHB7* was fused into the expression vector pCold TF. The HIS and HIS-FveHB7 proteins were induced using 0.1 mM IPTG at 16°C for 20 h in BL21(DE3) transit cells and then were purified by ProteinIso^®^ Ni-NTA Resinkit (TransGen). Biotin 3′-end-labeled DNA hot and mutant probes were synthesized or amplified using biotin 3′-end-labeled primers (Shangon, Shanghai, China). Biotin-labeled DNA was detected using the SDS-PAGE Gel Kit (Cowin, Jiangsu, China) and the chemiluminescence EMSA kit (Beyotime, Shanghai, China). EMSA images were captured using a Tanon-5200 imaging system (Tanon, Shanghai, China). All primers and probes used are listed in Table S2. The experiments were repeated three times independently.

### *In vitro* protein degradation assay

Total protein was extracted from the shoot tips of FveDAD2-RNAi plants and WT (YW) plants. The extracts were incubated with bacterially expressed FveSMXL7-GST recombinant protein in the presence of either DMSO (solvent control) or the proteasome inhibitor MG132 for 30 minutes. Western blot analysis was performed using an anti-GST antibody to monitor the degradation of FveSMXL7-GST, with actin serving as the loading control for the plant protein extracts.

### Hormone quantification

‘YW’ and FveDAD2-RNAi plants were cultivated in pots containing perlite and irrigated every 3 days with a solution of half-strength Hoagland. Shoot tips of WT and transgenic plants were collected separately as samples (about 0.5 g each) for phytohormone content determination, when differences in the number of new branch crowns began to appear between WT and transgenic plants. Samples were immediately frozen in liquid nitrogen and homogenized to a fine powder. ABA was extracted and quantified according to technical support (Wuhan MetWare Biotechnology Co., Ltd, Wuhan, China). The ABA was extracted by the Ultra Performance Liquid Chromatography (ExionLC™ AD, America) and Tandem Mass Spectrometry (SCIEX QTRAP 6500+, America) (LC–MS/MS) systems. ABA was quantified by the external standard technique and is presented as ng/g fresh weight (FW).

### Plant phenotypic characterization statistics

*FveDAD2* transgenic plants and ‘YW’ plants with uniform growth conditions were selected, and the following data were investigated and counted. The number of branch crowns: a single branch crown with an independent growing point is counted as one branch crown; plant height (cm): the vertical height from the base of the strawberry stem to the highest point of the plant; number of leaves: the strawberry leaf was counted as a single leaf when all the ternary leaves of the strawberry were expanded; leaf area (cm^2^): the product of the length and width of the central leaf of the third ternately compound leaf from the center of the strawberry outward was calculated; the single fruit weight (g): the weight of a primary fruit; and the total soluble solids content of a primer fruit. To quantify the plant phenotype, A–C indicate significant differences among the plants (Prism 8.0.1 Duncan’s MRT, *n* = 6, *P* < 0.01).

### Accession numbers

TAIR: AtD14 (AT3G03990.1), AtSMXL7 (AT2G29970.1), AtHB7 (AT2G46680.1); GDR: FveDAD2 (FvYW_2g33160), FveKAI2 (FvH4_1g17110), FveD14 (FvH4_1g05320), FveSMXL7 (FvH4_7g24540), FveHB7 (FvH4_7g17320), and FveABA8'OH1 (FvH4_2g39460); NCBI: FveDAD2 (XP_004290965.1), FveKAI2 (XP_004287973.1), FveD14 (XP_004287076.1), SlDAD2(XP_004238093.1), PhDAD2(sp|J9U5U9.1|), PsRMS3(sp|A0A109QYD3.1|), ZmD14(XP_008660429.1), SbD14(XP_002468316.1), OsD53 (NP_001410055.1), FveSMXL7 (XP_011469920.1), and FveHB7 (XP_004306630.1).

## Supplementary Material

Web_Material_uhaf250

## Data Availability

All relevant data can be found within the manuscript and its supporting materials.
