# Supplementary material for: FveDAD2 negatively regulates branch crowns by affecting abscisic acid metabolism through FveHB7 in woodland strawberry
Source: Hortic Res. 2025 Sep 17;13(1):uhaf250. doi: 10.1093/hr/uhaf250 (PMC12856502; doi:10.1093/hr/uhaf250)
Supplement: Web_Material_uhaf250 [file web_material_uhaf250.zip › Figure S2.pdf]

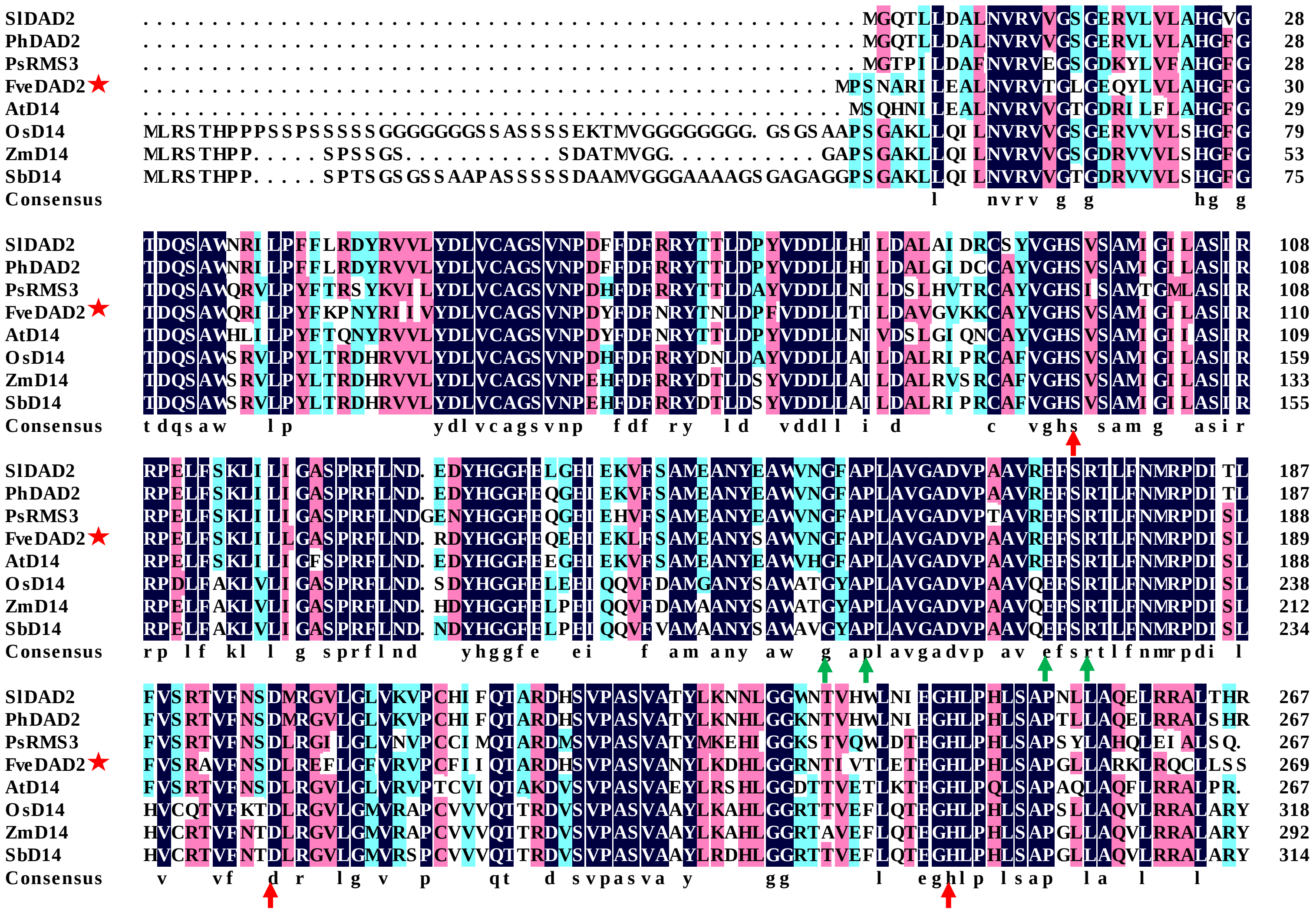

SlDAD2 .......................................MGQTLLDALNVRVVGSGERVLVLAHGVG 28
PhDAD2 .......................................MGQTLLDALNVRVVGSGERVLVLAHGFG 28
PsRMS3 .......................................MGTPILDAFNVRVEGSGDKYLVFAHGFG 28
FveDAD2 .......................................MPSNARILEALNVRVTGLGEQYLVLAHGFG 30
AtD14 .......................................MSQHNILEALNVRVVGTGDRILFLAHGFG 29
OsD14 MLRSTHPPPSSPSSSSSGGGGGGGSSASSSEKTMVGGGGGGGG.GSGSAAPSGAKLLQILNVRVVGSGERVVVLSHGFG 79
ZmD14 MLRSTHPP.....SPSSGS...........SDATMVGG...........GAPSGAKLLQILNVRVVGSGDRVVVLSHGFG 53
SbD14 MLRSTHPP.....SPTSGSGSSAAPASSSSDAAMVGGGAAAAGSGAGAGGPSGAKLLQILNVRVVGTGDRVVVLSHGFG 75
Consensus l nvrv g g hg g
SlDAD2 TDQSAWNRILPFFLRDYRVVLYDLVCAGSVNPDFFDFRRYTTLDPYVDDLLHILDALAIDRCSYVGHSVSAMIGILASIR 108
PhDAD2 TDQSAWNRILPFFLRDYRVVLYDLVCAGSVNPDFFDFRRYTTLDPYVDDLLHILDALGIDCCAYVGHSVSAMIGILASIR 108
PsRMS3 TDQSAWQRVLPYFTRSYKVILYDLVCAGSVNPDHFDFRRYTTLDAYVDDLLNILDSLHVTRCAYVGHSISAMTGMLASIR 108
FveDAD2 TDQSAWQRILPYFKPNYRIIVYDLVCAGSVNPDYFDFNRYTNLDPFVDDLLTILDAVGVKKCAYVGHSVSAMIGILASIR 110
AtD14 TDQSAWHLILPYFTQNYRVVLYDLVCAGSVNPDYFDFNRYTTLDPYVDDLLNIVDSLGIQNCAYVGHSVSAMIGIIASIR 109
OsD14 TDQSAWSRVLPYLTRDHRVVLYDLVCAGSVNPDHFDFRRYDNLDAYVDDLLAILDALRIPRCAFVGHSVSAMIGILASIR 159
ZmD14 TDQSAWSRVLPYLTRDHRVVLYDLVCAGSVNPEHFDFRRYDTLDSYVDDLLAILDALRVSRCAFVGHSVSAMIGILASIR 133
SbD14 TDQSAWSRVLPYLTRDHRVVLYDLVCAGSVNPEHFDFRRYDTLDSYVDDLLAILDALRIPRCAFVGHSVSAMIGILASIR 155
Consensus t dqsaw lp ydl vcagsvnp f df ry l d vddll i d c vghs sam g asir
SlDAD2 RPELFSKLILIGASPRFLND.EDYHGGFELGEIEKVFSAMEANYEAWVNGFAPLAVGADVPAAVREFSRTLFNMRPDITL 187
PhDAD2 RPELFSKLILIGASPRFLND.EDYHGGFEQGEIEKVFSAMEANYEAWVNGFAPLAVGADVPAAVREFSRTLFNMRPDITL 187
PsRMS3 RPELFSKLILIGASPRFLNDGENYHGGFEQGEIEHVFSAMEANYEAWVNGFAPLAVGADVPTAVREFSRTLFNMRPDISL 188
FveDAD2 RPELFSKLILLGASPRFLND.RDYHGGFEQEEIEKLFSAMEANYSAWVNGFAPLAVGADVPAAVREFSRTLFNMRPDISL 189
AtD14 RPELFSKLILIGFSPRFLND.EDYHGGFEEGEIEKVFSAMEANYEAWVHGFAPLAVGADVPAAVREFSRTLFNMRPDISL 188
OsD14 RPDLFAKLVLIGASPRFLND.SDYHGGFELEEIQQVFDAMGANYSAWATGYAPLAVGADVPAAVQEFSRTLFNMRPDISL 238
ZmD14 RPELFAKLVLIGASPRFLND.HDYHGGFELPEIQQVFDAMAANYSAWATGYAPLAVGADVPAAVQEFSRTLFNMRPDISL 212
SbD14 RPELFAKLVLIGASPRFLND.NDYHGGFELPEIQQVFVAMAANYSAWAVGYAPLAVGADVPAAVQEFSRTLFNMRPDISL 234
Consensus rp lf kl l g sprflnd yhggfe ei f am any aw g apl avgadvp av efsrtlfnmrpdi l
SlDAD2 FVSRTVFNSDMRGVLGLVKVPCHIFQTARDHSVPASVATYLKNNLGGWNTVHWLNIEGHLPHLSAPNLLAQELRRALTHR 267
PhDAD2 FVSRTVFNSDMRGVLGLVKVPCHIFQTARDHSVPASVATYLKNHLGGKNTVHWLNIEGHLPHLSAPTLLAQELRRALSHR 267
PsRMS3 FVSRTVFNSDLRGILGLVNVPCCIMQTARDMSVPASVATYMKEHIGGKSTVQWLDTEGHLPHLSAPSYLAHQLEIALSQ. 267
FveDAD2 FVSRAVFNSDLREFLGFVRVPCFIIQTARDHSVPASVANYLKDHLGGRNTIVTLETEGHLPHLSAPGLLARKLRQCLLSS 269
AtD14 FVSRTVFNSDLRGVLGLVRVPTCVIQTAKDVSVPASVAEYLRSHLGGDTTVETLKTEGHLPQLSAPAQLAQFLRRALPR. 267
OsD14 HVCQTVFKTDLRGVLGMVRAPCVVVQTTRDVSVPASVAAYLKAHLGGRTTVEFLQTEGHLPHLSAPSLLAQVLRRALARY 318
ZmD14 HVCRTVFNTDLRGVLGMVRAPCVVVQTTRDVSVPASVAAYLKAHLGGRTAVEFLQTEGHLPHLSAPGLLAQVLRRALARY 292
SbD14 HVCRTVFNTDLRGVLGMVRSPCVVVQTTRDVSVPASVAAYLRDHLGGRTTVEFLQTEGHLPHLSAPGLLAQVLRRALARY 314
Consensus v vf d r lg v p qt d svpasva y gg l eghl p lsap la l l
