# Supplementary material for: FveDAD2 negatively regulates branch crowns by affecting abscisic acid metabolism through FveHB7 in woodland strawberry
Source: Hortic Res. 2025 Sep 17;13(1):uhaf250. doi: 10.1093/hr/uhaf250 (PMC12856502; doi:10.1093/hr/uhaf250)
Supplement: Web_Material_uhaf250 [file web_material_uhaf250.zip › Figure S5.pdf]

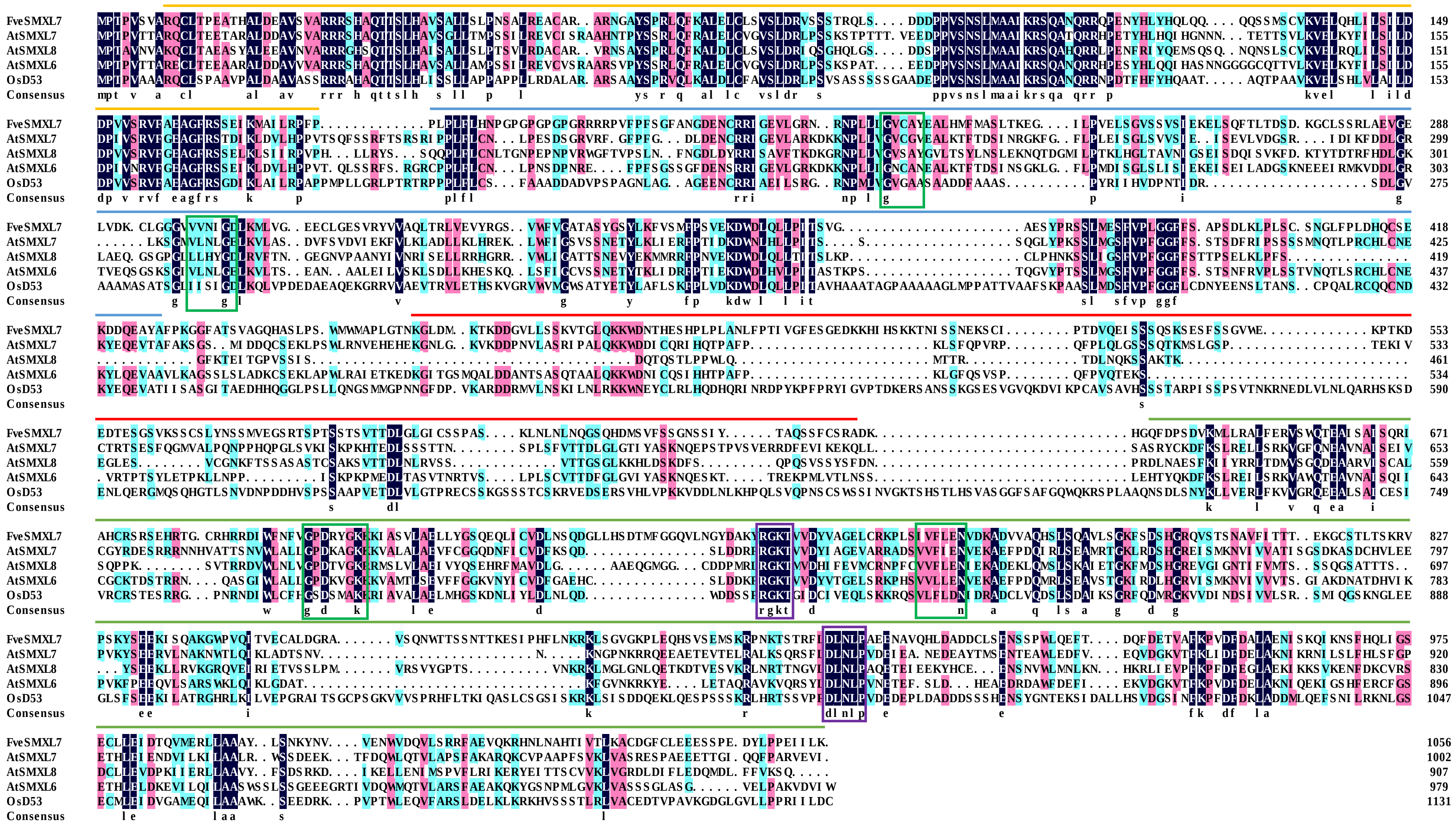
FveSMXL7
AtSMXL7
AtSMXL8
AtSMXL6
OsD53
Consensus
