# Supplementary material for: FveDAD2 negatively regulates branch crowns by affecting abscisic acid metabolism through FveHB7 in woodland strawberry
Source: Hortic Res. 2025 Sep 17;13(1):uhaf250. doi: 10.1093/hr/uhaf250 (PMC12856502; doi:10.1093/hr/uhaf250)
Supplement: Web_Material_uhaf250 [file web_material_uhaf250.zip › Figure S8.pdf]

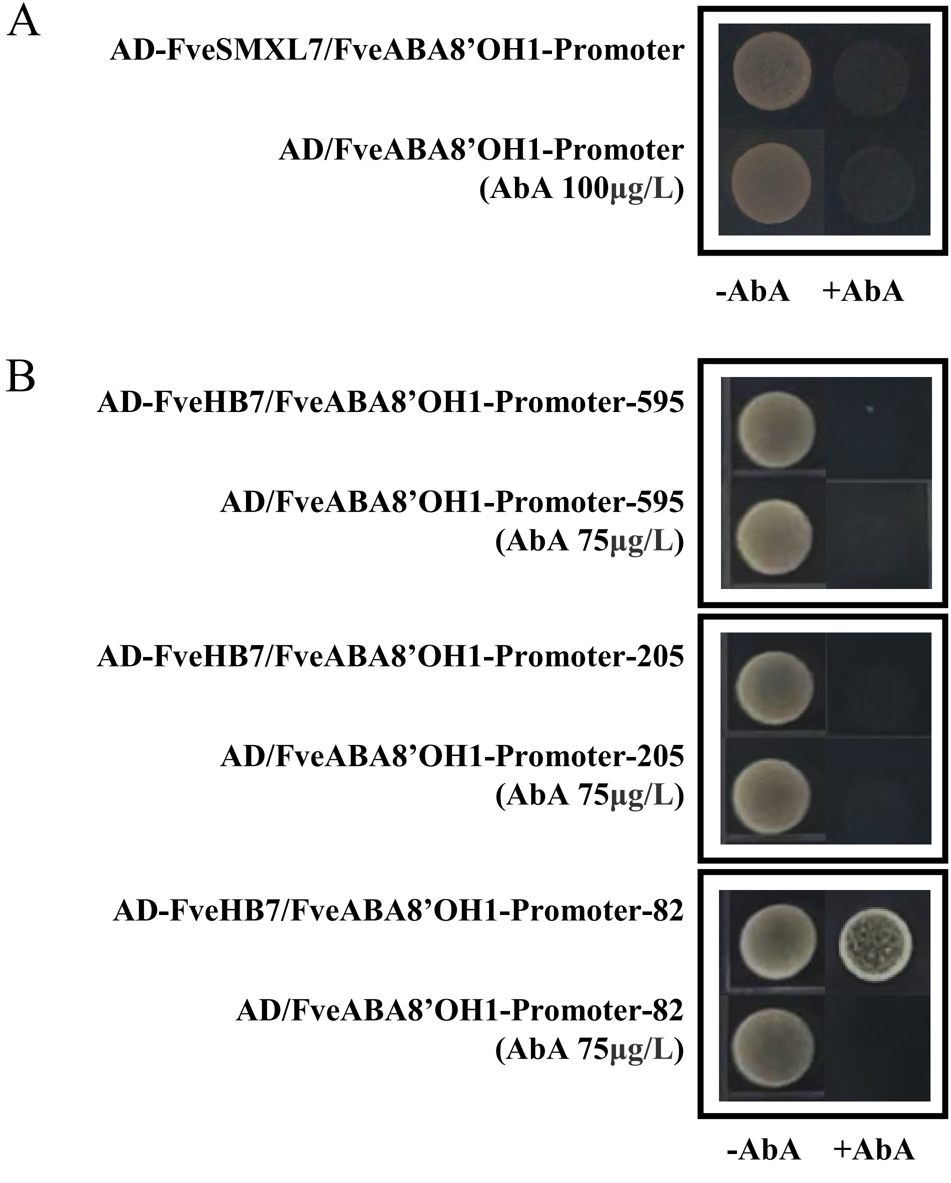
A
AD-FveSMXL7/FveABA8’OH1-Promoter
AD/FveABA8’OH1-Promoter
(AbA 100µg/L)
-AbA
+AbA
B
AD-FveHB7/FveABA8’OH1-Promoter-595
AD/FveABA8’OH1-Promoter-595
(AbA 75µg/L)
AD-FveHB7/FveABA8’OH1-Promoter-205
AD/FveABA8’OH1-Promoter-205
(AbA 75µg/L)
AD-FveHB7/FveABA8’OH1-Promoter-82
AD/FveABA8’OH1-Promoter-82
(AbA 75µg/L)
-AbA
+AbA
