# Supplementary material for: FveDAD2 negatively regulates branch crowns by affecting abscisic acid metabolism through FveHB7 in woodland strawberry
Source: Hortic Res. 2025 Sep 17;13(1):uhaf250. doi: 10.1093/hr/uhaf250 (PMC12856502; doi:10.1093/hr/uhaf250)
Supplement: Web_Material_uhaf250 [file web_material_uhaf250.zip › Supplemental Figure1-9 FveDAD2 regulates branch crowns through ABA.docx]

## upplemental Figure


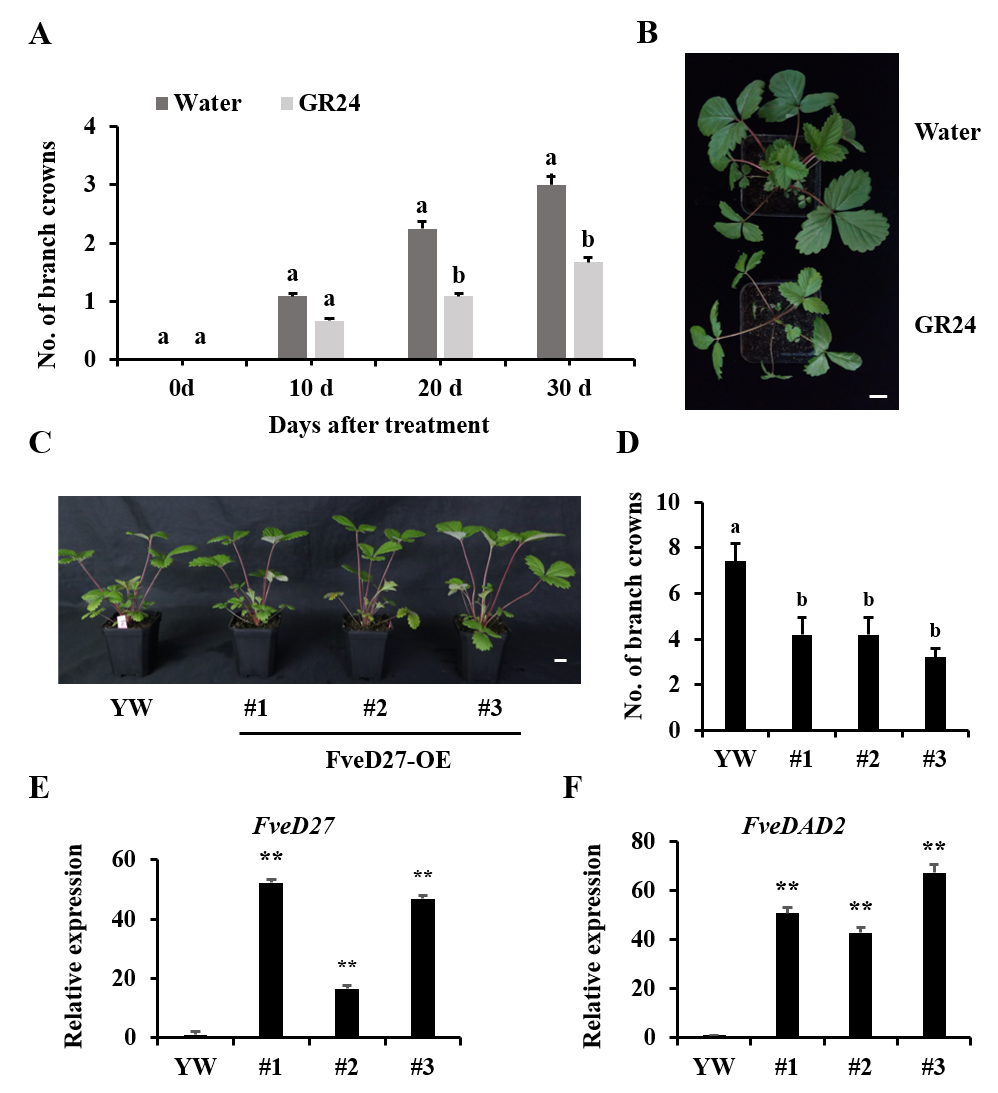


### Supplemental Fig. S1 Strigolactone affects the number of branch crowns in the woodland strawberry.

**A** The effect of 5 μM GR24 on the branch crowns of strawberry. 0 d: the day of the first treatment application; 10 d, 20 d, 30 d: 10, 20, 30 days of continuous application. Values are means ± SD (n = 5), a, b indicate significant differences between plants (Prism 8.0.1, Duncan MRT, *P* < 0.05). **B** Phenotypic images of woodland strawberry treated with GR24 for 30d, Bar = 1cm. **C** Phenotypic images of the ‘YW’ and FveD27-OE lines during the vegetative stage (70 d old), Bar = 1cm. **D** The number of branch crowns in the ‘YW’ and FveD27-OE lines. Values are means ± SD (n = 6), A, B indicate significant differences between plants (Prism 8.0.1, Duncan MRT, *P* < 0.01). **E, F** Analysis of relative expression levels of *FveD27* and *FveDAD2* by qRT-PCR in ‘YW’ and FveD27-OE plants. Values are mean ± SD of three independent experiments with three biological replicates (DPS, Duncan’s MRT, ***P* < 0.01).


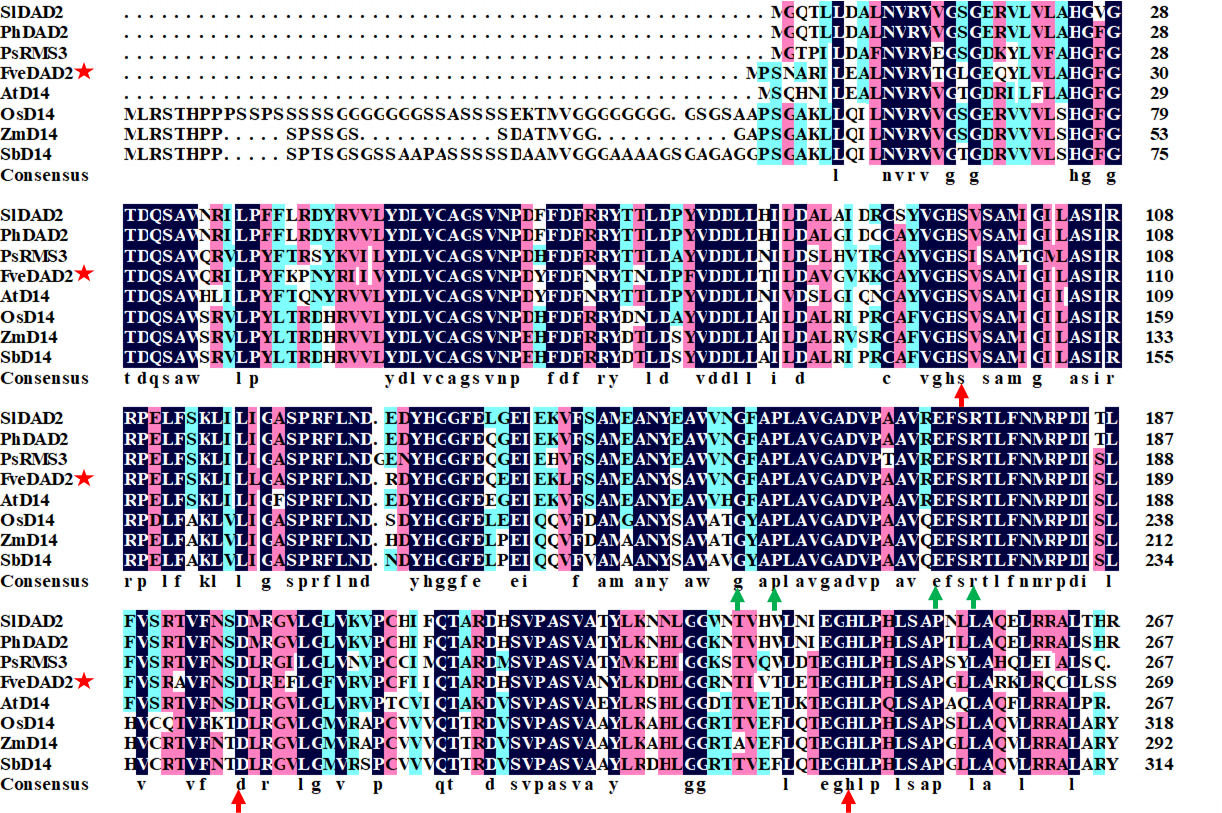


### Supplemental Fig. S2 Amino acid sequence comparison of FveDAD2 homologous proteins.

The amino acid sequence alignment of FveDAD2 and homologous proteins from the monocots and the dicots using DNAMAN. Catalytic triad (AtD14: Ser97, Asp218, His247; FveDAD2: Ser98, Asp199, His248; red arrow), and crucial amino acids for identifying downstream components (AtD14: Gly159, Pro162, Glu175, Ser177; FveDAD2: Gly160, Pro163, Glu176, Ser178; green arrow). The red pentagram represents FveDAD2.


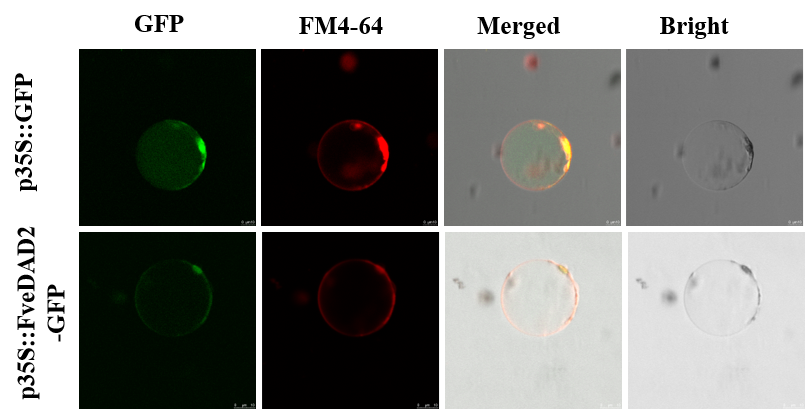


### Supplemental Fig. S3 Subcellular localization of FveDAD2 in the *Arabidopsis* protoplast.

The pro35S::FveDAD2-GFP fusion protein was co-localized to the nucleus, endoplasmic reticulum, and cytoplasm (Fm4-64: Membrane tissue localization markers).


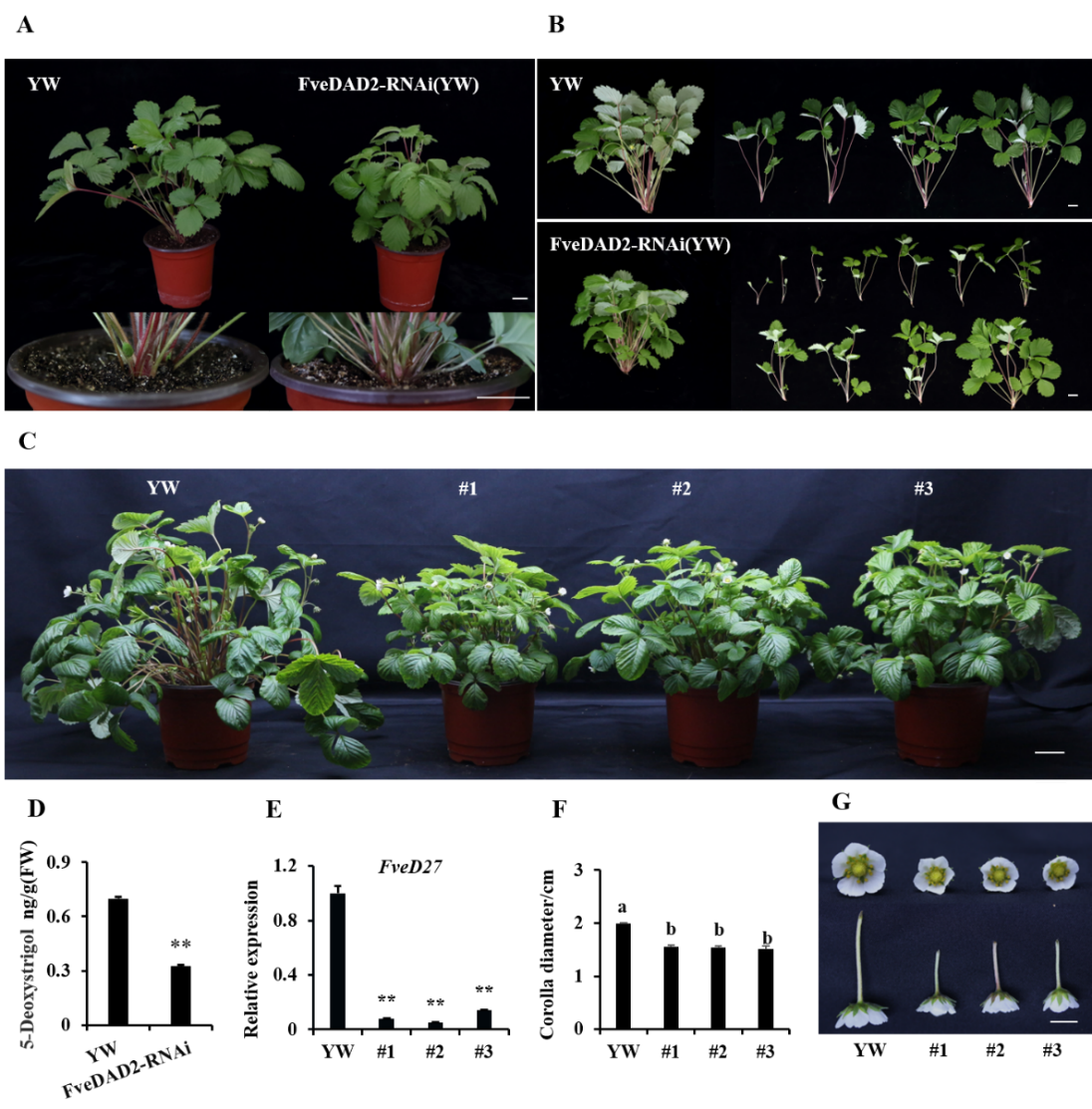


### Supplemental Fig. S4 The detailed phenotypes of FveDAD2-RNAi lines and the YW during the reproductive stage.

**A** The branch crowns of FveDAD2-RNAi lines and the YW during the early reproductive stage. Bar = 2 cm. **B** The detailed differences of branch crowns in FveDAD2-RNAi and YW. **C** The phenotypes of FveDAD2-RNAi lines and the YW during the reproductive stage. Bar = 5 cm. **D** 5-Deoxystrigol levels in the shoot tips of FveDAD2-RNAi lines and YW (DPS, Duncan’s MRT, ***P* < 0.01). **E** Analysis of relative expression levels of *FveD27* by qRT-PCR in YW and FveDAD2-RNAi plants. Values are mean ± SD of three independent experiments with three biological replicates (DPS, Duncan’s MRT, ***P* < 0.01). **F** The quantification of the corolla diameter. Values are Mean ± SD (*n* = 10), a and b indicate significant differences among the plants (DPS, Duncan’s MRT, *P* < 0.05). **G** The phenotypes of flower in the FveDAD2-RNAi lines and the YW. Bar = 1 cm.


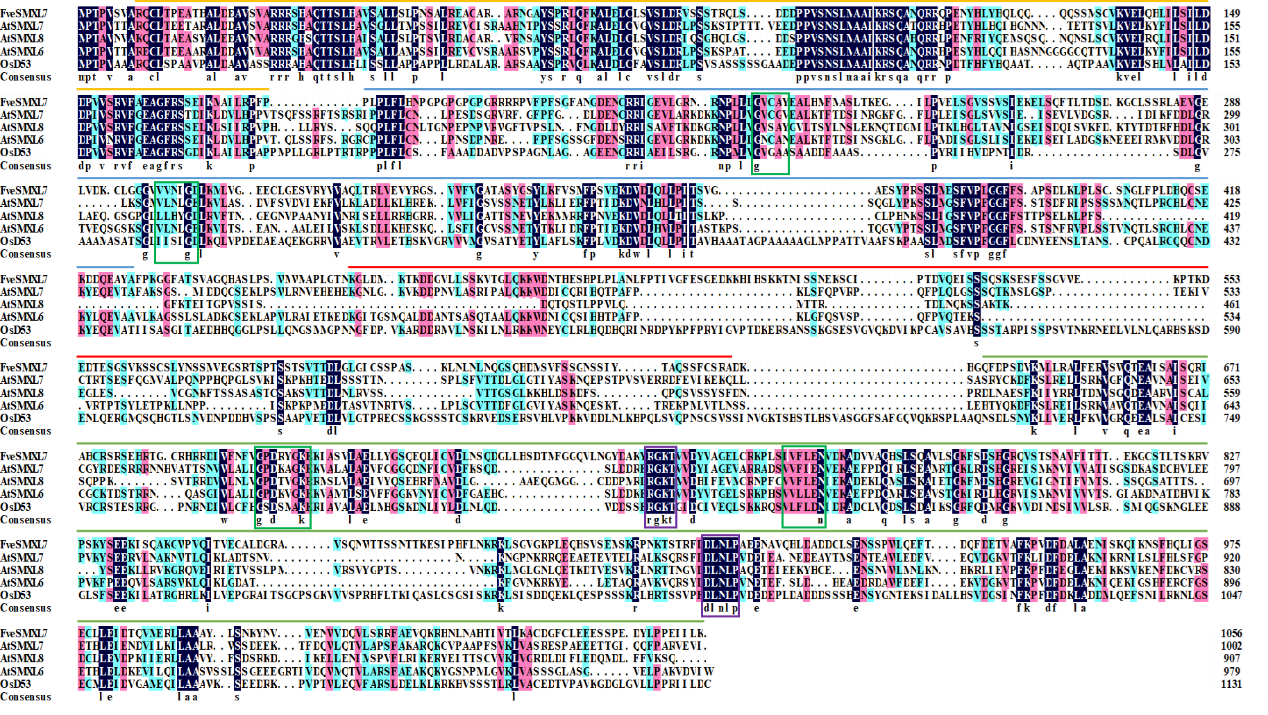


### Supplemental Fig. S5 SMXL7 amino acid sequence comparison.

The amino acid sequence alignment of FveSMXL7, AtSMXL7, AtSMXL6, AtSMXL8 and OsD53 using DNAMAN multiple amino acid alignment (Yellow line: N-terminal domain, blue line: D1 ATPase domain, red line: M domain, green line: D2 ATPase domain, green box: Walker A (WA) and Walker B, purple box: the conserved motifs RGKT and LDLNL).


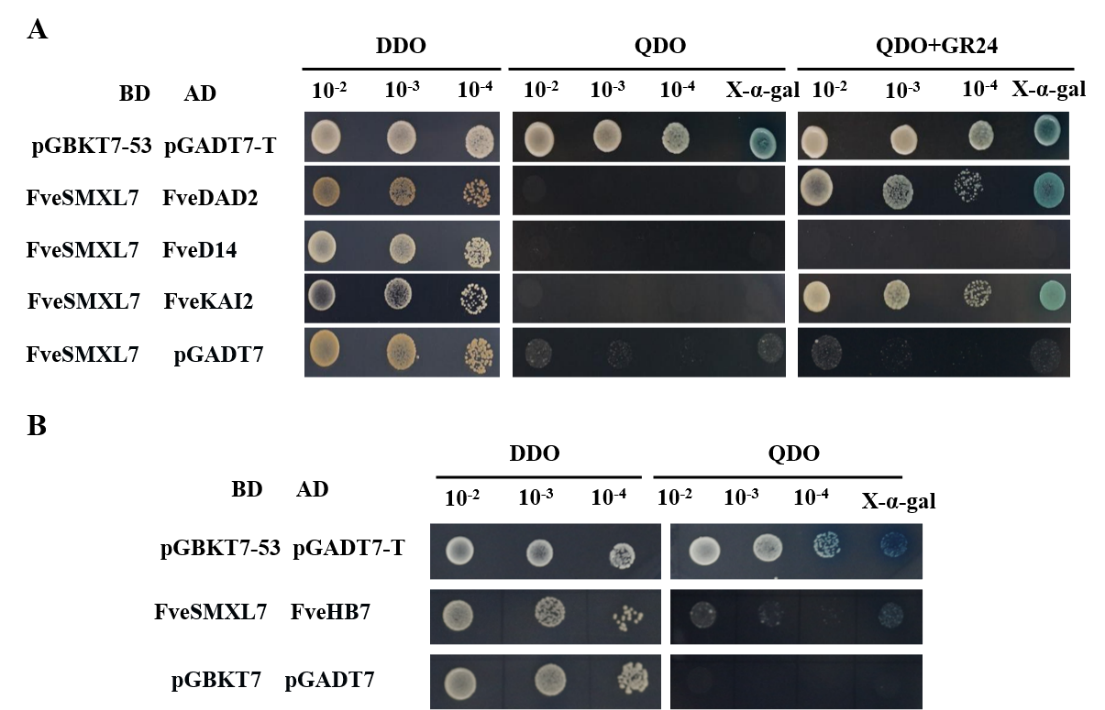


### Supplemental Fig. S6 FveSMXL7-related yeast two-hybrid assay.

**A** A Y2H assay was conducted to demonstrate the interaction between FveDAD2/FveD14/FveKAI2 and FveSMXL7 in the presence of GR24. **B** A Y2H assay was conducted to demonstrate the interaction between FveHB7 and FveSMXL7.


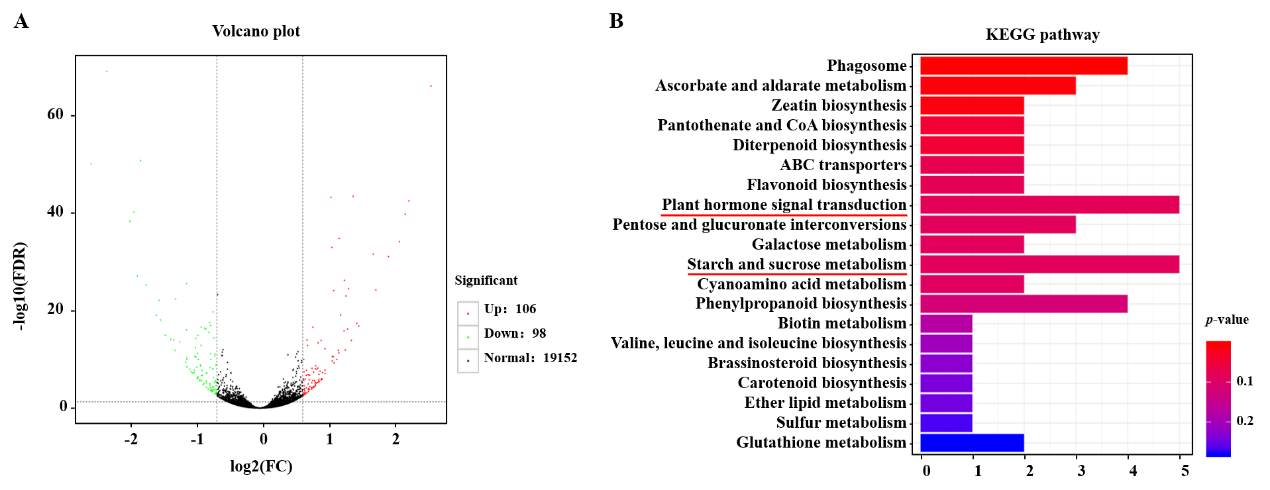


### Supplemental Fig. S7 RNA-seq analysis of shoot tips in FveDAD2-RNAi transgenic lines and YW.

**A** Differential expression volcano plot in the transcriptome data (|Fold Change| ≥ 1.5 and FDR < 0.05). Each point in the differential expression volcano plot represents a gene, and the horizontal coordinate indicates the logarithmic value of the fold difference in expression between two samples for a particular gene; the vertical coordinate indicates the negative logarithmic value of the statistical significance of the change in gene expression. The green points: down-regulated genes, the red points: up-regulated genes, and the black points: non-differentially genes. **B** KEGG enrichment of genes in the transcriptome analysis. The horizontal coordinate for GeneNum indicates the number of differential genes annotated in the entry and the vertical coordinate for each pathway entry. The colors of the bars represent the *p*-value of the hypergeometric test. The red line indicates the pathway where DEGs are enriched to a greater extent.


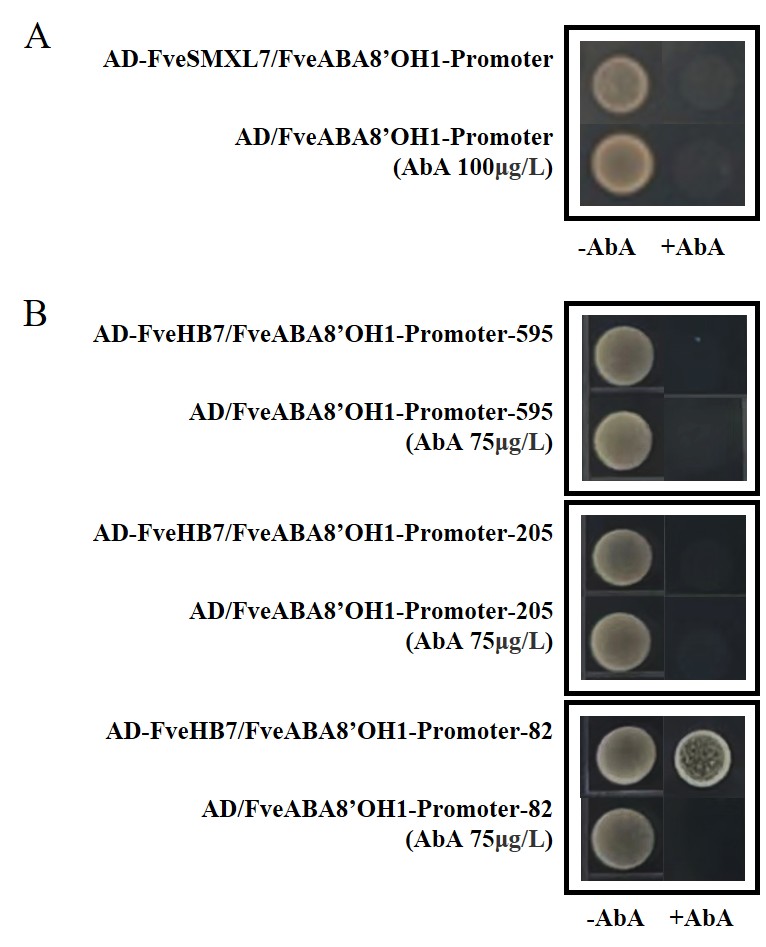


### Supplemental Fig. S8 The Y1H assay of FveSMXL7/FveHB7 binding to the FveABA8'OH1 promoter.

**A** The Y1H assay indicates that FveSMXL7 does not bind to the promoter of *FveABA8'OH1*. **B** There are three ATGAT sites (-595 bp, -205 bp, and -82 bp) in the promoter of *FveABA8'OH1*. The segmented Y1H assay indicates that FveHB7 binds to the ATGAT (-82bp) in the promoter of *FveABA8'OH1*. The baseline concentration of aureobasidin A (AbA) used was 75 μg/L. The negative controls utilized were the empty vector and the *FveHB7*/*FveABA8'OH1* promoter (-595, -205, -82).


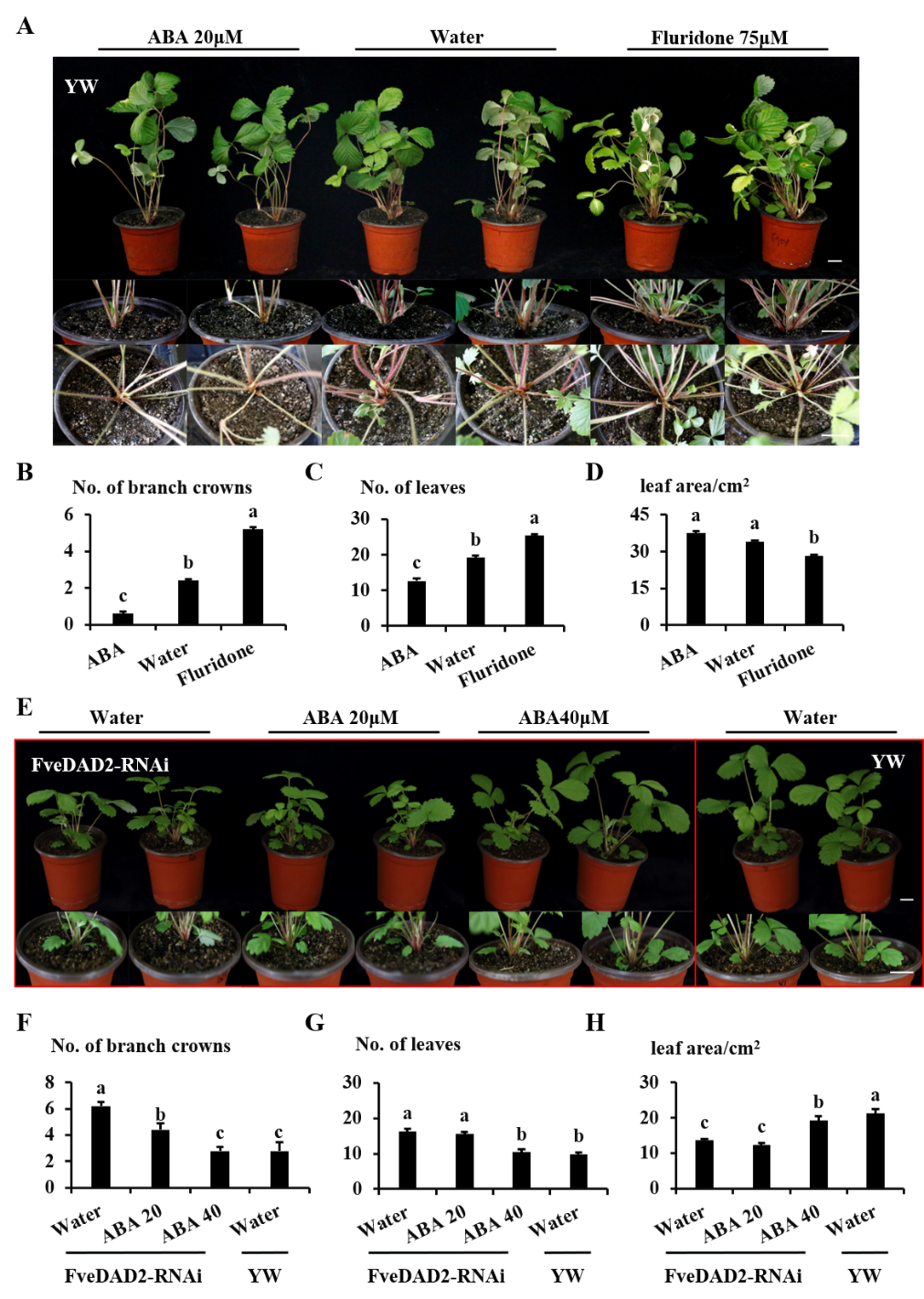


### Supplemental Fig. S9 Effect of ABA and ABA inhibitor on branch crowns and leaves in YW and FveDAD2-RNAi plants.

**A** Phenotypes of YW after one month of treatment with ABA and fluridone (an inhibitor of ABA). Bar = 2cm. **B** Number of branch crowns, **C** number of leaves, **D** and leaf area after one month of ABA and fluridone treatment in YW. Values are Mean ± SD (*n* = 5), a–c indicate significant differences among the plants (DPS, Duncan’s MRT, *P* < 0.05). **E** Phenotypes of FveDAD2-RNAi after one month of treatment with ABA. Bar = 2cm. Number of branch crowns **F**, number of leaves **G**, and leaf area **H** after one month of ABA treatment in FveDAD2-RNAi and the YW. Values are Mean ± SD (*n* = 5), a–c indicate significant differences among the plants (DPS, Duncan’s MRT, *P* < 0.05).
