# Supplementary material for: FveDAD2 negatively regulates branch crowns by affecting abscisic acid metabolism through FveHB7 in woodland strawberry
Source: Hortic Res. 2025 Sep 17;13(1):uhaf250. doi: 10.1093/hr/uhaf250 (PMC12856502; doi:10.1093/hr/uhaf250)
Supplement: Web_Material_uhaf250 [file web_material_uhaf250.zip › Supplemental Table 1-2 FveDAD2 regulates branch crowns through ABA.docx]

## Supplemental Table

### Supplemental Table S1. The DEGs are related to hormones and sugar through the NR annotation

| **Gene ID** | **Regulated** | **NR_annotation** | **Classifications** |
| --- | --- | --- | --- |
| FvH4_2g39460 | up | abscisic acid 8&apos; -hydroxylase 1 isoform X1 | Hormone-ABA |
| FvH4_2g34420 | up | abscisic acid 8&apos; -hydroxylase 4-like |  |
| FvH4_6g10740 | up | abscisic acid receptor PYL4 |  |
| FvH4_7g31810 | down | probable protein phosphatase 2C 51 |  |
| FvH4_7g17320 | down | homeobox-leucine zipper protein ATHB-12-like |  |
| FvH4_7g32570 | down | homeobox-leucine zipper protein ATHB-12-like |  |
| FvH4_2g37300 | up | ABC transporter G family member 14 |  |
| FvH4_2g37120 | down | histidine kinase 5 |  |
| FvH4_7g17340 | up | auxin-induced protein X15-like | Hormone-Auxin |
| FvH4_1g18170 | down | auxin-binding protein ABP19a-like |  |
| FvH4_7g24200 | up | auxin transporter-like protein 2 |  |
| FvH4_3g33580 | down | lysine-specific demethylase REF6-like | Hormone-BR |
| FvH4_6g46500 | down | cytochrome P450 85A isoform X1 | Hormone-CTK |
| FvH4_6g24620 | down | cytokinin dehydrogenase 1 |  |
| FvH4_3g03260 | up | cytokinin dehydrogenase 6-like |  |
| FvH4_2g34020 | down | ethylene-responsive transcription factor RAP2-1-like | Hormone-ETH |
| FvH4_6g50260 | down | gibberellin-regulated protein 14-like | Hormone-GA |
| FvH4_4g36510 | down | gibberellin 2-beta-dioxygenase 8-like |  |
| FvH4_7g28670 | down | gibberellin 20 oxidase 1 |  |
| FvH4_2g24370 | down | gibberellin receptor GID1B-like |  |
| FvH4_2g33160 | down | uncharacterized protein LOC101311534 | Hormone-SL |
| FvH4_7g24540 | up | uncharacterized protein LOC105353070 |  |
| FvH4_2g25960 | up | probable pectinesterase/pectinesterase inhibitor 7 | Sugar |
| FvH4_3g43500 | up | beta-glucosidase 12-like |  |
| FvH4_4g30310 | down | beta-glucosidase 40 |  |
| FvH4_3g41930 | down | probable galactinol--sucrose galactosyltransferase 1 |  |
| FvH4_6g23550 | down | probable galactinol--sucrose galactosyltransferase 2 |  |
| FvH4_1g06350 | down | probable pectinesterase 53 |  |
| FvH4_3g23310 | up | GDP-L-galactose phosphorylase 2 |  |
| FvH4_4g27370 | up | GDP-L-galactose phosphorylase 2 |  |
| FvH4_3g07880 | up | UDP-glycosyltransferase 74F2-like |  |
| FvH4_3g02500 | down | glucose-6-phosphate/phosphate translocator 1, chloroplastic |  |
| FvH4_5g21090 | up | mannose-6-phosphate isomerase 2-like |  |
| FvH4_3g04100 | down | probable trehalose-phosphate phosphatase C isoform X2 |  |

### Supplemental Table S2. All primer information is included in this study

| **Prime name** | **Sequence** |
| --- | --- |
| FveDAD2-RNAiL-F | GCTCTAGACAAACCTAACTACCGCATCAT |
| FveDAD2-RNAiL-R | TCCCCCGGGTCCATCGCCGAGAACAA |
| FveDAD2-RNAiR-F | GGGGTACCTCCATCGCCGAGAACAA |
| FveDAD2-RNAiR-R | CGAGCTCCAAACCTAACTACCGCATCAT |
| FveDAD2-GFP-F | ATATGCCCGTCGACCCCGGGGGTACCATGCCCAGCAACGCCAGGAT |
| FveDAD2-GFP-R | TCCTCGCCCTTGCTCACCATGGATCCCGACGACAACAGACACTGGC |
| Fve26S-F | TAACCGCATCAGGTCTCCAA |
| Fve26S-R | CTCGAGCAGTTCTCCGACAG |
| FveDAD2-QF | CAACGCCAGGATCTTAGAGG |
| FveDAD2-QR | TCGAAGTAATCGGGGTTGAC |
| FveD27-QF | GCCGTCTTTACCACCA |
| FveD27-QR | GCATTCCCATTGTATCCT |
| FveSMXL7-QF | GTTTCCGTCGGTAGAGAAAGAC |
| FveSMXL7-QR | CAACATAACTCACCTGGACCTC |
| FveHB7-QF | AGGTCCGAATCTGAGGGGAA |
| FveHB7-QR | ATCGGAGTTCAATCTGCCCC |
| FveABA8OH1-QF | TGTTTGGGGATGAACATGGCTA |
| FveABA8OH1-QR | TTGATACGAGTCACAGTCACCG |
| FveDAD2-BD-F | TGCATATGGCCATGGAGGCCGAATTCATGCCCAGCAACGCCAGGAT |
| FveDAD2-BD-R | TGCGGCCGCTGCAGGTCGACGGATCCCTACGACGACAACAGACACT |
| FveSMXL7-BD-F | TGCATATGGCCATGGAGGCCGAATTCATGCCTACGCCAGTTTCTGT |
| FveSMXL7-BD-R | TGCGGCCGCTGCAGGTCGACGGATCCTCACTTTAGTATGATTTCGG |
| FveSMXL7-AD-F | ATATGGCCATGGAGGCCAGTGAATTCATGCCTACGCCAGTTTCTGT |
| FveSMXL7-AD-R | ATCTGCAGCTCGAGCTCGATGGATCCTCACTTTAGTATGATTTCGG |
| FveDAD2-NE-F | TGGCGCGCCACTAGTGGATCCATGCCCAGCAACGCCAGGAT |
| FveDAD2-NE-R | CCCGGGAGCGGTACCCTCGAGGTCGACCGACGACAACAGACACT |
| FveSMXL7-CE-F | TGGCGCGCCACTAGTGGATCCATGCCTACGCCAGTTTCTGT |
| FveSMXL7-CE-R | CCCGGGAGCGGTACCCTCGAGGTCGACCTTTAGTATGATTTCGG |
| FveHB7-AD-F | ATATGGCCATGGAGGCCAGTGAATTCATGTTTGAGTCAGACCAAGT |
| FveHB7-AD-R | ATCTGCAGCTCGAGCTCGATGGATCCTCAAGACCAAAAATTCCACC |
| PA-FveHB7-F | AAATGATGAATTGAAAAGCCTGCATATGCTAGTACGTTCTC |
| PA-FveHB7-R | AGATCCCCGGGTACCGAGCTCCCAATTAAGCTGGTGGTTTC |
| PA-FveABA8’OH1-F | AAATGATGAATTGAAAAGCTTATCGATTGGGATGGTGGTGC |
| PA-FveABA8’OH1-R | AGATCCCCGGGTACCGAGCTCAAATGAGGTCCCAAGGTAGA |
| pRI101-FveSMXL7-OE-F | CAAGTTCTTCACTGTTGATACATATGATGCCTACGCCAGTTTCTGTA |
| pRI101-FveSMXL7-OE-R | GAGTTGTTGATTCAGAATTCGGATCCTCACTTTAGTATGATTTCGG |
| ProFveHB7-luc-F | CGACGGTATCGATAAGCTTCGTGAGGACATTCCTCTACC |
| ProFveHB7-luc-R | CTCTAGAACTAGTGGATCCCCAATTAAGCTGGTGGTTTC |
| FveHB7-OE-F | CACTGTTGATACATATGCCCGTCGACATGTTTGAGTCAGACCAAGT |
| FveHB7-OE-R | GAGTTGTTGATTCAGAATTCGGATCCTCAAGACCAAAAATTCCACC |
| ProFveA81-luc-F | CGACGGTATCGATAAGCTTTCCTCCACTCGAATCTTCTG |
| ProFveA81-luc-R | CTCTAGAACTAGTGGATCCAAATGAGGTCCCAAGGTAGA |

### Supplemental Table S2. All primer information is included in this study (continued)

| **Prime name** | **Sequence** |
| --- | --- |
| ProHB7-GUS-F | CCAGTGCCAAGCTTGCATGCCTGCAGCGTGAGGACATTCCTCTACC |
| ProHB7-GUS-R | TTTTGTGTGATTGTGATGTATCTAGACCAATTAAGCTGGTGGTTTC |
| ProABA8’OH1-GUS-F | CCAGTGCCAAGCTTGCATGCCTGCAGTCCTCCACTCGAATCTTCTG |
| ProABA8’OH1-GUS-R | TTTTGTGTGATTGTGATGTATCTAGAAAATGAGGTCCCAAGGTAGA |
| At18S-F | GAGAAGTTACTCCGCAACCT |
| At18S-R | GAATGATGCGTCGCCAGCACAAAGG |
| AtABA8’OH1-QF | GTGTTCCTGTCTACGACCGA |
| AtABA8’OH1-QR | TGTGATGGTGTTGAGGAGCA |
| FveSMXL7-GST-F | TTCCAGGGGCCCCTGGGATCCATGCCTACGCCAGTTTCTGT |
| FveSMXL7-GST-R | GTCACGATGCGGCCGCTCGAGTCACTTTAGTATGATTTCGG |
| FveHB7-HIS-F | GTATCGAAGGTAGGCATATGATGTTTGAGTCAGACCAAGT |
| FveHB7-HIS-R | GCAGAGATTACCTATCTAGATCAAGACCAAAAATTCCACC |
| FveHB7-Biop-F | AAGACGTAAACAAATTGCTTATAACAAAGTGTAATTTTCTCTGGTGA |
| FveHB7-Biop-R | TCACCAGAGAAAATTACACTTTGTTATAAGCAATTTGTTTACGTCTT |
| FveHB7-probe-F | AAGACGTAAACAAATTGCTTATAACAAAGTGTAATTTTCTCTGGTGA |
| FveHB7-probe-R | TCACCAGAGAAAATTACACTTTGTTATAAGCAATTTGTTTACGTCTT |
| FveHB7-mBiop-F | AAGACGTAAACAAATTGCTTGCGGTGGAGTGTAATTTTCTCTGGTGA |
| FveHB7-mBiop-R | TCACCAGAGAAAATTACACTCCACCGCAAGCAATTTGTTTACGTCTT |
| FveABA8’OH1-43-Biop-F | AAATTGTTCAAGAATTCATAATGATAAAGCTACCGCACAAGTTTG |
| FveABA8’OH1-43-Biop-R | CAAACTTGTGCGGTAGCTTTATCATTATGAATTCTTGAACAATTT |
| FveABA8’OH1-43-probe-F | AAATTGTTCAAGAATTCATAATGATAAAGCTACCGCACAAGTTTG |
| FveABA8’OH1-43-probe-R | CAAACTTGTGCGGTAGCTTTATCATTATGAATTCTTGAACAATTT |
| FveABA8’OH1-43-mBiop-F | AAATTGTTCAAGAATTCATAGCAGCAAAGCTACCGCACAAGTTTG |
| FveABA8’OH1-43-mBiop-R | CAAACTTGTGCGGTAGCTTTGCTGCTATGAATTCTTGAACAATTT |
