# Supplementary figures and images for: FveDAD2 negatively regulates branch crowns by affecting abscisic acid metabolism through FveHB7 in woodland strawberry
Source: Hortic Res. 2025 Sep 17;13(1):uhaf250. doi: 10.1093/hr/uhaf250 (PMC12856502; doi:10.1093/hr/uhaf250)

**A**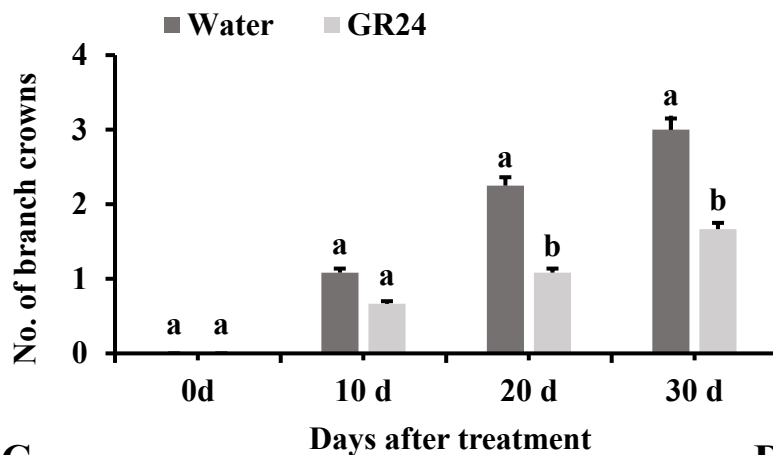**B**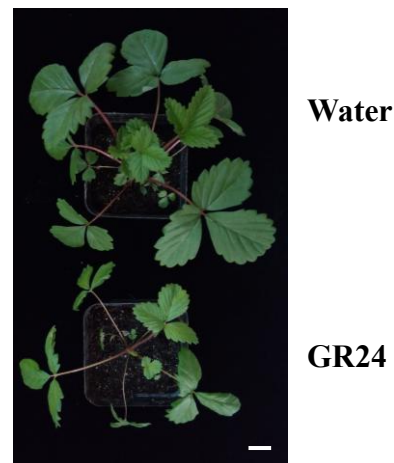**C**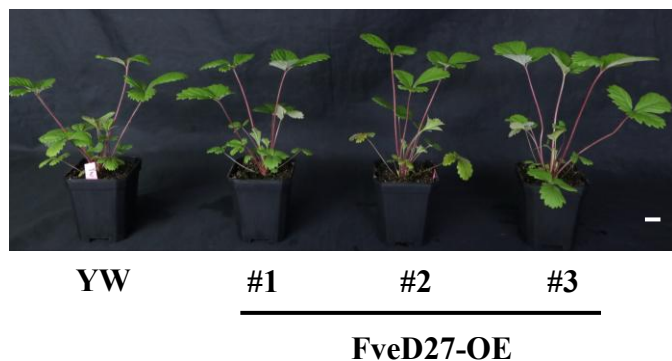**D**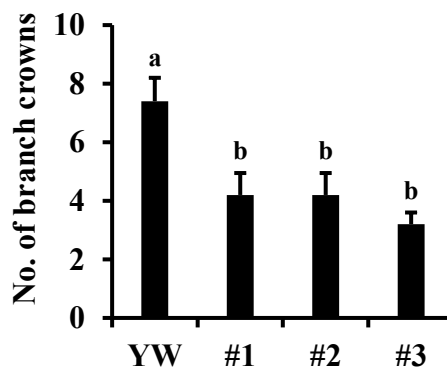**E**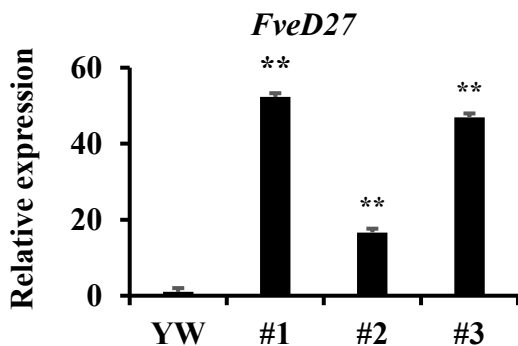**F**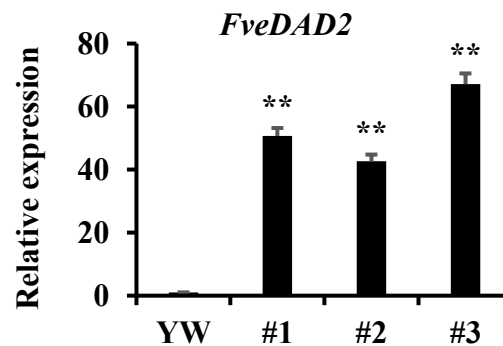

Supplement: Web_Material_uhaf250 [file web_material_uhaf250.zip › Figure S1.pdf]

**p35S::FveDAD2  
-GFP**

**p35S::GFP**

**GFP**

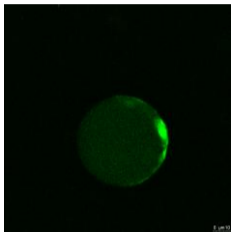

**FM4-64**

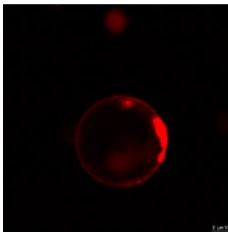

**Merged**

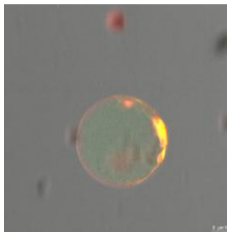

**Bright**

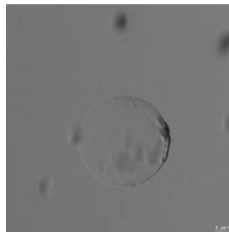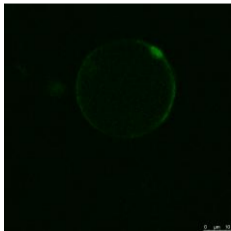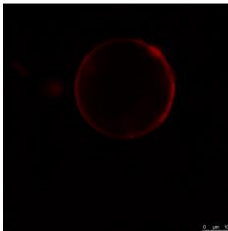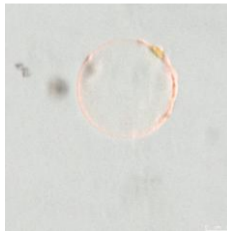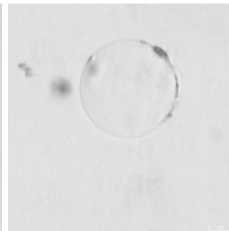

Supplement: Web_Material_uhaf250 [file web_material_uhaf250.zip › Figure S3.pdf]

**A**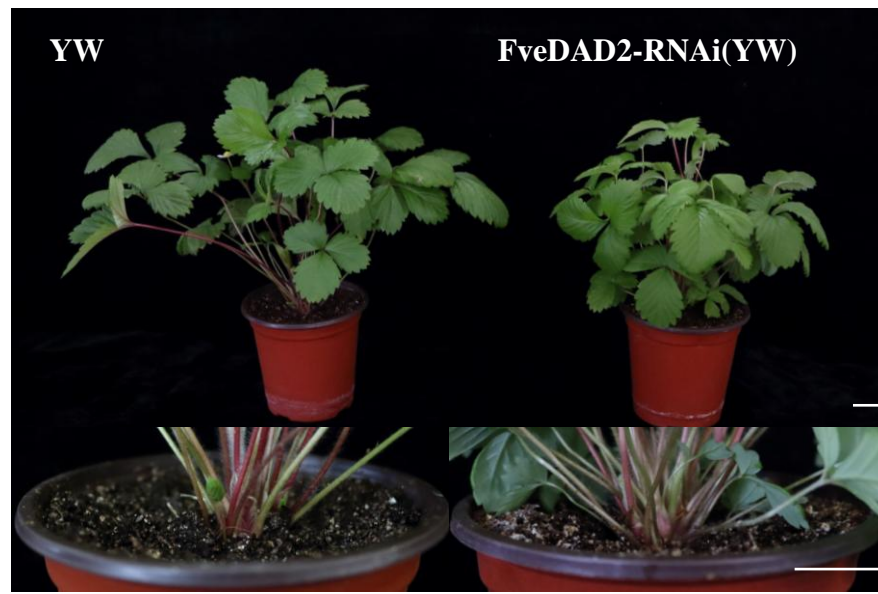**B**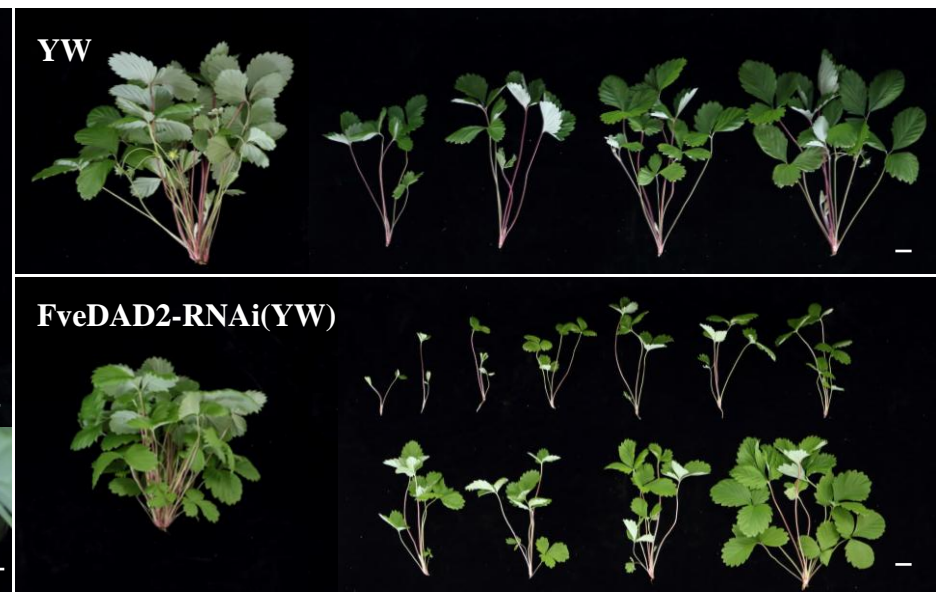**C**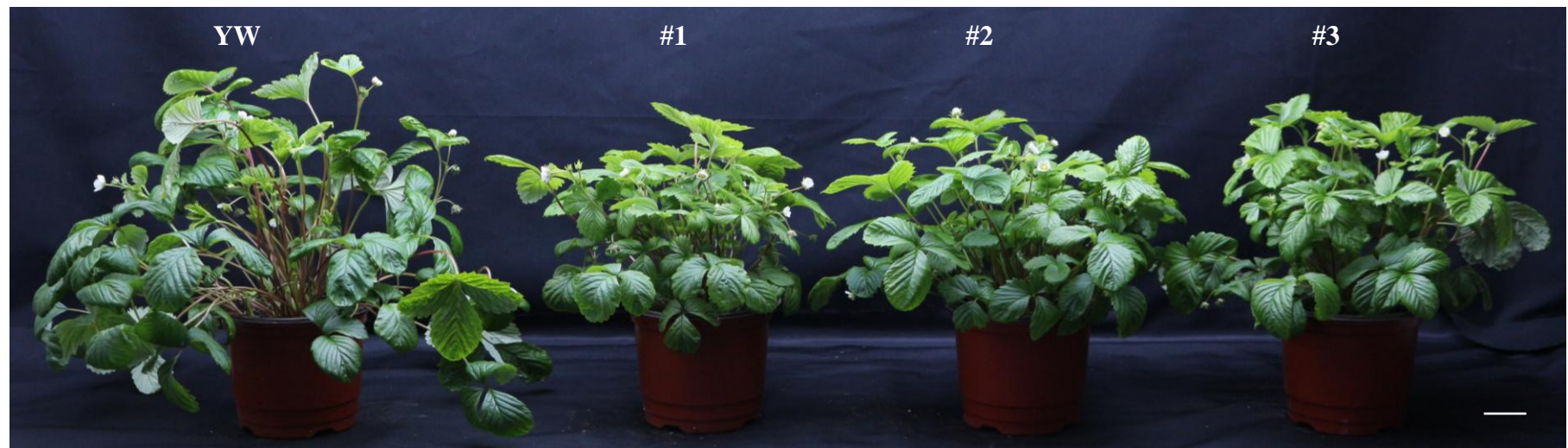**D**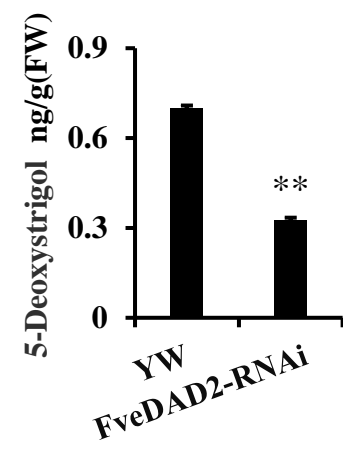**E**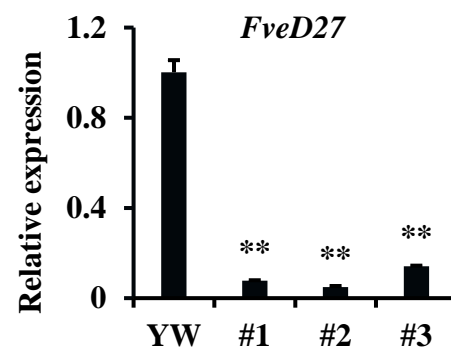**F**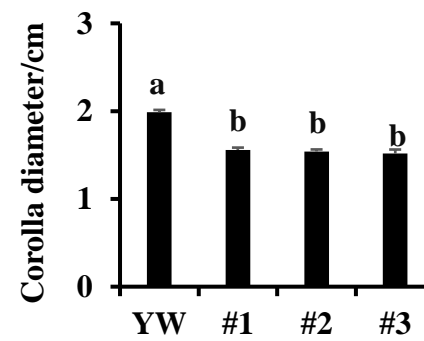**G**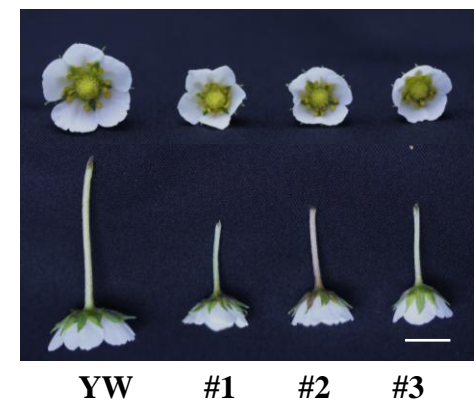

Supplement: Web_Material_uhaf250 [file web_material_uhaf250.zip › Figure S4.pdf]

**A**

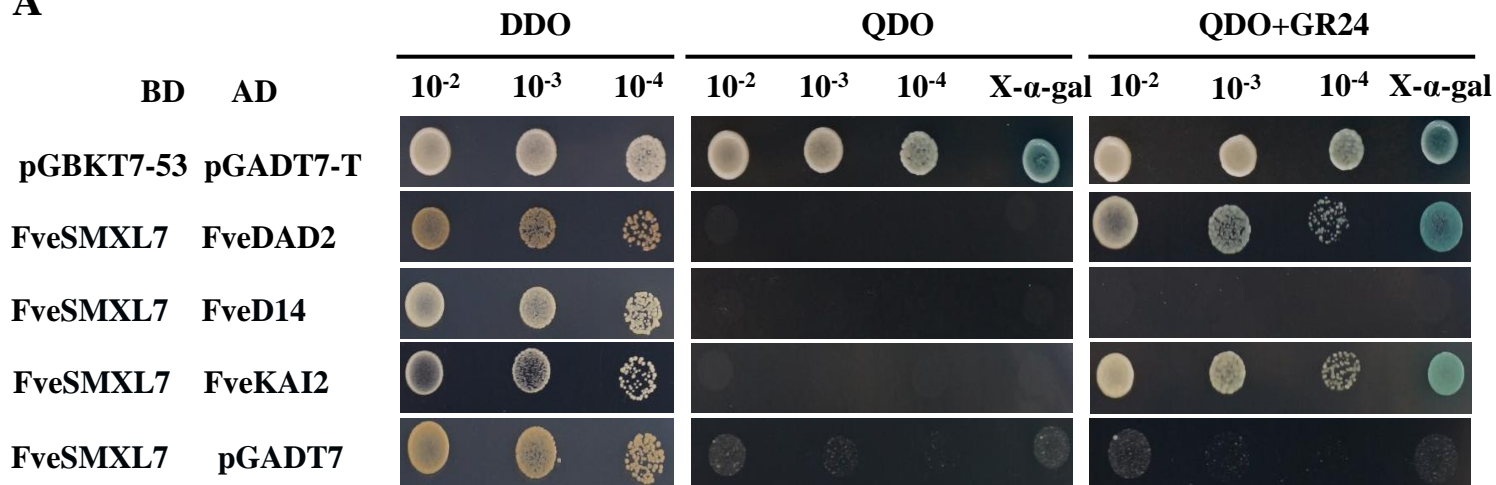

**B**

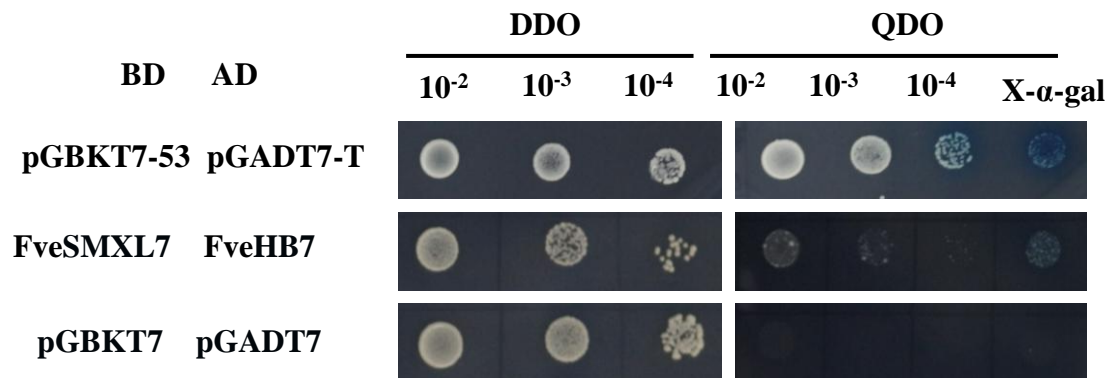

Supplement: Web_Material_uhaf250 [file web_material_uhaf250.zip › Figure S6.pdf]

**A****Volcano plot**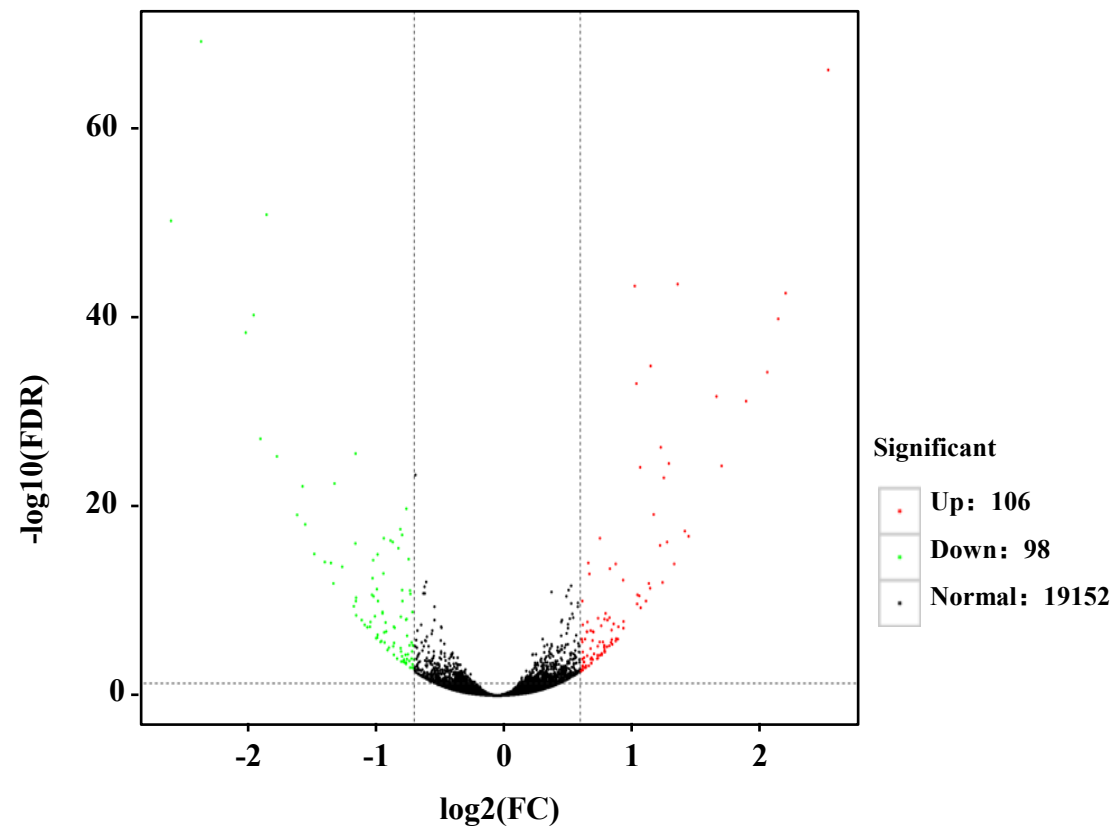**B****KEGG pathway**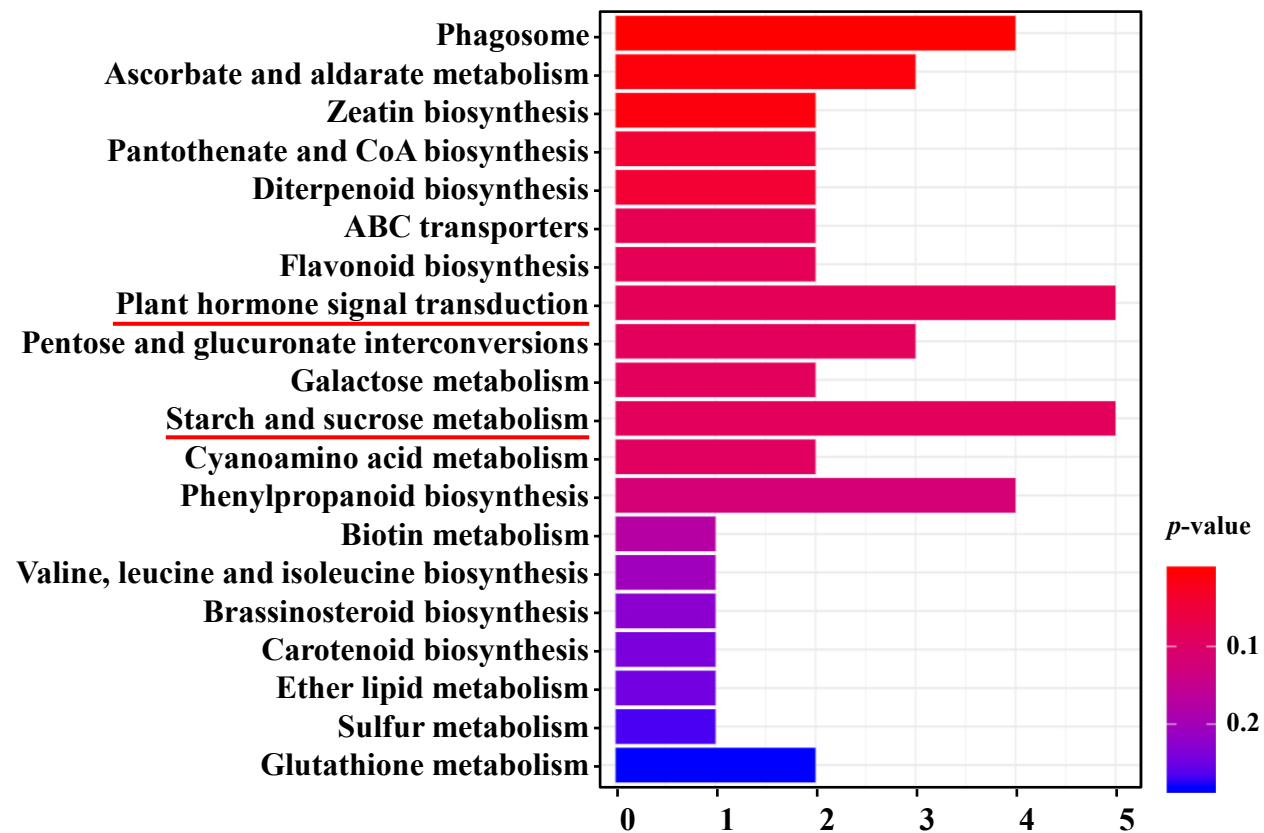

Supplement: Web_Material_uhaf250 [file web_material_uhaf250.zip › Figure S7.pdf]

**A**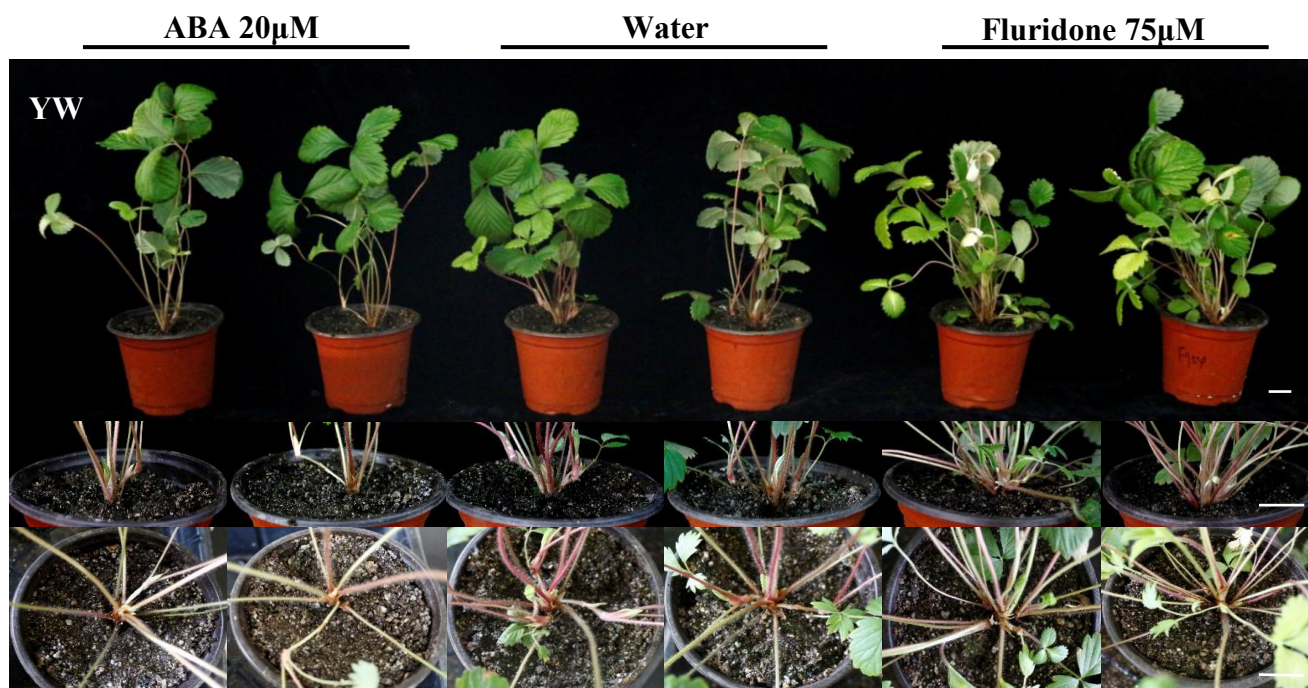**B**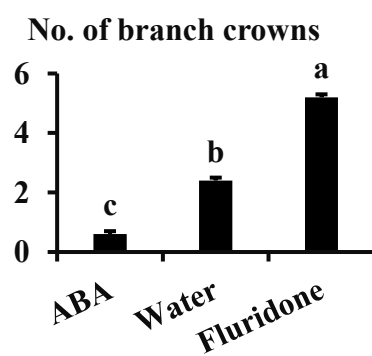**C**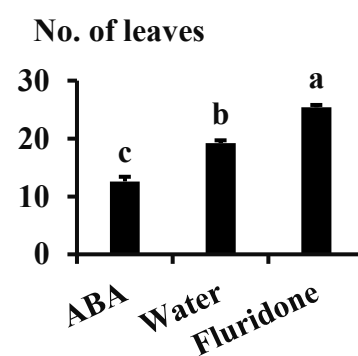**D**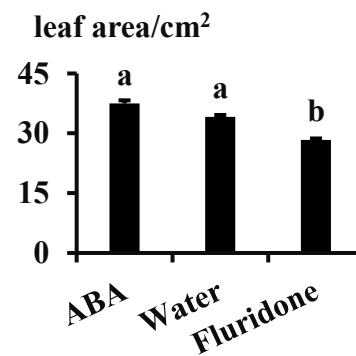**E**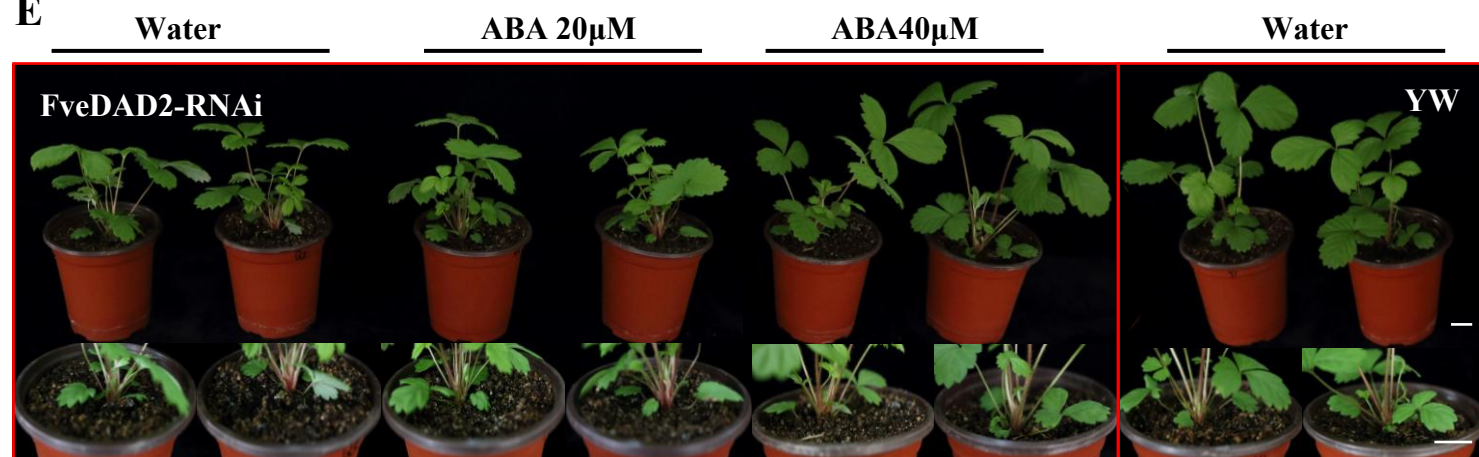**F**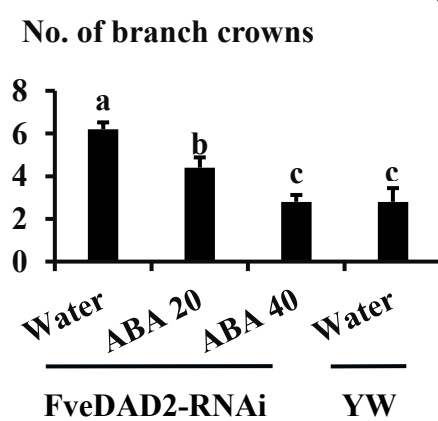**G**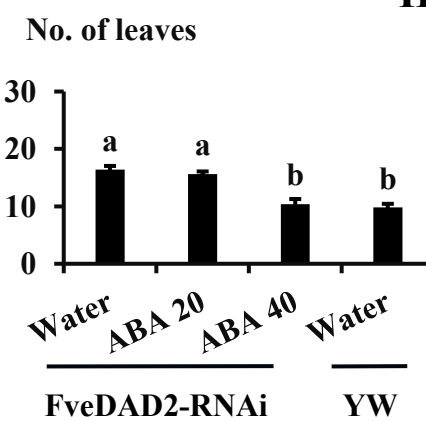**H**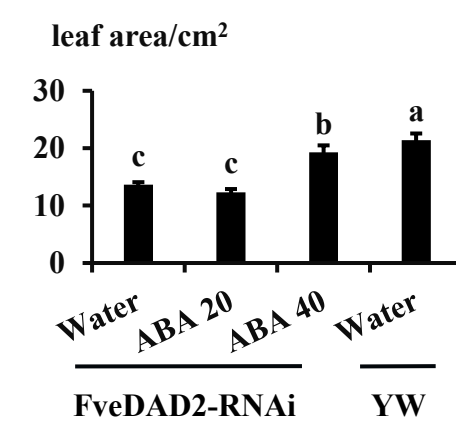

Supplement: Web_Material_uhaf250 [file web_material_uhaf250.zip › Figure S9.pdf]
